# Proteasomal subunit depletions differentially affect germline integrity in *C. elegans*

**DOI:** 10.3389/fcell.2022.901320

**Published:** 2022-08-17

**Authors:** Lourds Michelle Fernando, Cristina Quesada-Candela, Makaelah Murray, Caroline Ugoaru, Judith L. Yanowitz, Anna K. Allen

**Affiliations:** ^1^ Department of Biology, Howard University, Washington, DC, United States; ^2^ Magee-Womens Research Institute and Department of Obstetrics, Gynecology, and Reproductive Sciences, University of Pittsburgh School of Medicine, Pittsburgh, PA, United States; ^3^ Departments of Developmental Biology, Microbiology, and Molecular Genetics, The Hillman Cancer Center, University of Pittsburgh School of Medicine, Pittsburgh, PA, United States

**Keywords:** *C. elegans*, germ line, 19S regulatory particle, proteasome, meiosis

## Abstract

The 26S proteasome is a multi-subunit protein complex that is canonically known for its ability to degrade proteins in cells and maintain protein homeostasis. Non-canonical or non-proteolytic roles of proteasomal subunits exist but remain less well studied. We provide characterization of germline-specific functions of different 19S proteasome regulatory particle (RP) subunits in *C. elegans* using RNAi specifically from the L4 stage and through generation of endogenously tagged 19S RP lid subunit strains. We show functions for the 19S RP in regulation of proliferation and maintenance of integrity of mitotic zone nuclei, in polymerization of the synaptonemal complex (SC) onto meiotic chromosomes and in the timing of SC subunit redistribution to the short arm of the bivalent, and in turnover of XND-1 proteins at late pachytene. Furthermore, we report that certain 19S RP subunits are required for proper germ line localization of WEE-1.3, a major meiotic kinase. Additionally, endogenous fluorescent labeling revealed that the two isoforms of the essential 19S RP proteasome subunit RPN-6.1 are expressed in a tissue-specific manner in the hermaphrodite. Also, we demonstrate that the 19S RP subunits RPN-6.1 and RPN-7 are crucial for the nuclear localization of the lid subunits RPN-8 and RPN-9 in oocytes, further supporting the ability to utilize the *C. elegans* germ line as a model to study proteasome assembly real-time. Collectively, our data support the premise that certain 19S RP proteasome subunits are playing tissue-specific roles, especially in the germ line. We propose *C. elegans* as a versatile multicellular model to study the diverse proteolytic and non-proteolytic roles that proteasome subunits play *in vivo*.

## Introduction

The 26S proteasome is a ∼2.5 MDa multi-subunit protein complex that maintains cellular homeostasis by degrading old, misfolded, mistranslated, and/or regulatory proteins in cells in both the cytoplasm and the nucleus (Hanna and Finley, 2007; Pack et al., 2014; Bard et al., 2018; Marshall and Vierstra, 2019). Recent evidence shows that specific proteasome subunits play tissue specific and/or non-proteolytic roles in various organisms (Pispa et al., 2008; Bhat and Greer, 2011; Pispa et al., 2020). This includes roles in various cellular processes such as transcription, mRNA export, cell cycle regulation, and chromosome structure maintenance (Ferdous et al., 2002; Kwak et al., 2011; Seo et al., 2017; Gómez-H et al., 2019). Models such as yeast and mammalian cell lines are widely used to characterize proteasome function, however, these unicellular models have limitations in comprehensively understanding the wide range of roles that individual proteasome subunits might be playing in different tissues and developmental stages (Hochstrasser, 1996; Bai et al., 2019). Proper understanding of the assembly, structure, and function of the proteasome is crucial for understanding the pathology of diseases caused by irregular proteasome function, such as neurodegenerative diseases and cancer (Hanna and Finley, 2007; Hirano et al., 2008; Myeku et al., 2011; Kish-Trier and Hill, 2013; Saez and Vilchez, 2014; Schmidt and Finley, 2014; Maneix and Catic, 2016; Walerych et al., 2016).

**FIGURE 1:**
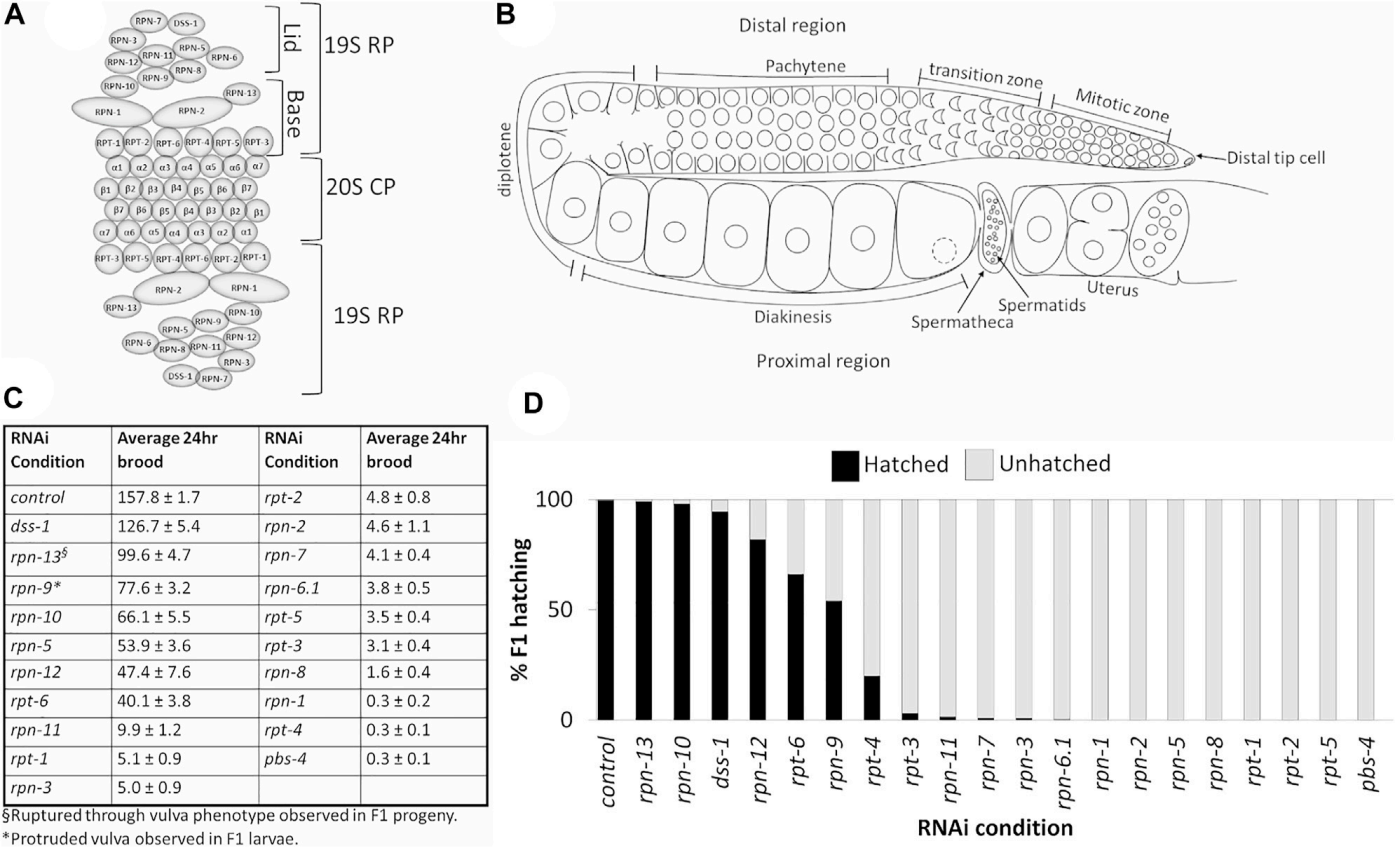
Depletion of 19S RP subunits of the 26S proteasome in *C. elegans* hermaphrodites causes reduced 24 h brood and/or embryonic lethality. **(A)** Schematic of eukaryotic 26S proteasome and its subunits. **(B)** Schematic of an adult *C. elegans* hermaphrodite germ line (one gonad arm). **(C)** Average 24 h brood of *C. elegans* hermaphrodites RNAi-depleted of either a control gene (*n* = 152), any of the 19 subunits of the 19S RP (*n* = 10–83), or a 20S CP subunit, PBS-4 (*n* = 36). Brood is shown ±SEM and calculated from at least three independent trials. All RNAi conditions compared to control exhibit a *p*-value < 0.0001. **(D)** Percent of hatched (black bars) and unhatched (grey bars) progeny of hermaphrodites treated with either *control(RNAi)* or the indicated *proteasome subunit(RNAi)*.

High resolution structural characterization of the 26S proteasome in human and yeast *via* cryo-electron microscopy and atomic modeling has revealed the structure of the eukaryotic proteasome at atomic level (Groll et al., 1997; Unno et al., 2002; Beck et al., 2012; Li et al., 2013; Huang et al., 2016). The mature 26S proteasome is composed of approximately 33 different, highly conserved protein subunits arranged into two 19S regulatory particles (RPs) capping one cylindrical 20S core particle (CP) (Figure 1A) (Kish-Trier and Hill, 2013). The 20S CP possesses the peptidase activity to degrade a protein substrate into smaller peptides, while the 19S RPs are responsible for recognizing, deubiquitinating and unfolding of polyubiquitinated substrates before importing substrates into the CP (Hanna and Finley, 2007; Finley, 2009). Each 19S RP is made up of two sub-complexes referred to as the lid and the base. The 19S RP lid is composed of non-ATPase subunits (Rpn3, Rpn5, Rpn6, Rpn7, Rpn8, Rpn9, Rpn11, Rpn12, and Sem1), while the base is composed of three non-ATPase subunits (Rpn1, Rpn2, and Rpn13) and six ATPase subunits (Rpt1, Rpt2, Rpt3, Rpt4, Rpt5, and Rpt6) (Kim, Yu and Cheng, 2011; Uprety et al., 2012). A final subunit, Rpn10, is thought to bridge the lid and base subcomplexes thus joining the two together (Bard et al., 2018). The *C. elegans* proteins comprising the 26S proteasome are diagrammed in Figure 1A and listed along with their human and yeast orthologs in Supplementary Table S1.

Assembly of the subunits to make a functional 26S proteasome is a highly conserved, multistep process. Yeast and mammalian studies have shown that the 26S proteasome can assemble in either the cytoplasm or the nucleus (Satoh et al., 2001; Yashiroda et al., 2008; Kaneko et al., 2009; Murata et al., 2009; Kish-Trier and Hill, 2013; Pack et al., 2014; Bai et al., 2019; Wendler and Enenkel, 2019). The 20S CP and 19S RP first assemble independently as subcomplexes in the cytoplasm and then either combine into the 26S in this compartment or are imported into the nucleus where they then assemble to form the mature 26S structure (Hirano et al., 2006; Kusmierczyk et al., 2008; Pack et al., 2014; Budenholzer et al., 2017; Marshall and Vierstra, 2019). The 20S CP subcomplex assembly is known to require the aid of non-proteasomal chaperone proteins, and nuclear localization sequences (NLSs) on the alpha subunits of the 20S CP aid in the nuclear import of the subcomplexes (Brooks et al., 2000; Hirano et al., 2006; Kusmierczyk et al., 2008; Budenholzer et al., 2017; Wu et al., 2018). The 19S RP lid and base subcomplexes assemble separately in the cytoplasm and either dock there on the assembled 20S CP to form the mature 26S proteasome in the cytoplasm, or are imported into the nucleus as separate modules before joining the 20S CP (Tanaka et al., 1990; Lehmann et al., 2002; Wendler et al., 2004). Previous research in yeast has identified assembly chaperones for the 19S RP base subcomplex and NLSs on two base subunits (yeast Rpt2 and Rpn2) aid in the nuclear import of the base (Wendler et al., 2004; Wendler and Enenkel, 2019). The yeast 19S RP lid subcomplex assembly consists first of the formation of Module 1 (Rpn5, Rpn6, Rpn8, Rpn9, and Rpn11) which then binds to lid particle 3 (Rpn3, Rpn7, and Sem1/Dss1) with Rpn12 serving as the linker (Budenholzer et al., 2017). Interestingly, no external factors or assembly chaperones have yet been identified that assist in 19S RP lid subcomplex assembly, nor do any of the lid subcomplex proteins have known NLS sequences which could aid in the nuclear import of the 19S lid (Isono et al., 2007; Budenholzer et al., 2020). Therefore, further studies are required to fill the gap in our understanding of nuclear import of the 19S lid subcomplex.

While the role of the proteasome as the protein degradation machine in eukaryotes is well characterized, recent findings have sparked an interest in non-canonical and tissue-specific roles of individual proteasome subunits and/or subcomplexes. In mammals, tissue-specific proteasomes, such as the immunoproteasome, thymoproteasome, and spermatoproteasome contain structural variations in specific proteasome subunits leading to their tissue specificity (Kish-Trier and Hill, 2013; Uechi et al., 2014; Gómez-H et al., 2019; Motosugi and Murata, 2019). Studies done in mammals and *C. elegans* show that the 19S RP lid subunit PSMD11/RPN-6.1 can regulate proteolytic activity of the proteasome modulating the production of the other proteasome subunits thus increasing or decreasing proteolytic activity of the proteasome (Vilchez and Boyer, 2012; Vilchez and Morantte, 2012; Lokireddy et al., 2015). *C. elegans* studies have also uncovered proteasome subunits that are specific for germline development and fertility (Shimada et al., 2006; Pispa et al., 2008; Fernando et al., 2020). RPN-10, RPN-12, and DSS-1 (RPN15/SEM1) were each shown to play specific roles in germline sex determination and oocyte development (Shimada et al., 2006; Pispa et al., 2008; Fernando et al., 2020).

Proper function of the 26S proteasome in the *C. elegans* hermaphrodite germ line is crucial for normal progression of meiosis and production of viable progeny (Glotzer et al., 1991; Lee and Schedl, 2010). The two germline arms of the nematode meet at a shared uterus. Each arm contains a distal mitotic pool of cells that enter meiosis as they move proximally (Figure 1B) (Hubbard and Greenstein, 2000; Hillers et al., 2017). The germline nuclei are open to the central rachis until the diakinesis stage when cellularization of the developing oocytes is completed (Pazdernik and Schedl, 2013). The oocytes briefly arrest at the diakinesis stage prior to maturation, ovulation, and completion of the meiotic divisions (Greenstein, 2005). Feeding L4 *C. elegans* hermaphrodites dsRNA against individual 19S RP proteasome subunits results in F1 progeny lethality for most of the 19S RP subunits, the exceptions being RPN-9, RPN-10, RPN-12, DSS-1, and RPT-6 (Takahashi et al., 2002; Shimada et al., 2006; Pispa et al., 2008; Fernando et al., 2020). Despite the impact on embryonic viability, the effect of 19S RP subunit depletion on the reproductive capabilities of the RNAi-treated hermaphrodite mothers has not been examined. Here we report fertility defects observed in *C. elegans* hermaphrodites RNAi-depleted of individual 19S RP subunits starting from the L4 stage. Our study includes testing of 19S RP subunits that were not part of a 2002 study that reported the embryonic lethality effect of RNAi depletion of various of the 26S proteasomal subunits (Takahashi et al., 2002).

Recently our labs separately characterized previously unknown roles for the proteasome in the germ line (Allen et al., 2014; Ahuja et al., 2017; Fernando et al., 2020). We reported interactions between specific 19S RP subunits with a major meiotic kinase, WEE-1.3; we also described synaptonemal complex (SC) defects upon impairment of the 20S proteasome (Allen et al., 2014; Ahuja et al., 2017; Fernando et al., 2020)*.* Here, we have embarked on a more detailed analysis of individual proteasomal subunit function in both the distal and proximal germ line of the *C. elegans* hermaphrodite. *C. elegans* is a powerful genetic model whose optical transparency enables the observation of biological processes in real-time and the determination of the subcellular localization of fluorescently tagged proteins of interest during any stage of the *C. elegans* life cycle. To help elucidate individual proteasome subunit functions in the germ line, we began endogenously tagging 19S RP lid subunits with GFP or OLLAS, and present novel tissue-specific expression of RPN-6.1 and genetic requirements for the nuclear localization of lid subunits RPN-8 and RPN-9 in the *C. elegans* oocyte. We propose *C. elegans* as a versatile multicellular model to study the diverse proteolytic and non-proteolytic roles proteasome subunits play *in vivo* in specific tissues and cell types.

## Materials and methods

### Strains

All strains were maintained at 20°C on standard MYOB or NGM plates seeded with OP50 unless mentioned otherwise (Brenner, 1974). Bristol strain N2 was used as the wild-type strain. Other strains used in this study are included in Supplementary Table S2.

### Strain generation

Strains in this study were generated using CRISPR/Cas9 genome editing technology following the direct delivery method developed by Paix et al. (2015). The Co-CRISPR method using *unc-58* or *dpy-10* was performed to screen for desired edits (Arribere et al., 2014). Specificity of the crRNAs were determined using UCSC genome browser and http://crispr.mit.edu/. ApE plasmid editor was used for sequence analysis to select PAM sites and primer designs. The edits were confirmed using PCR. At least two independent strains were generated for each edit (except N-terminal GFP tagged RPN-7 for which only one strain was generated) and the resulting edited strains backcrossed with wild type (N2) at least five times and sequenced before being utilized.

GFP tags were generated by inserting Superfolder GFP sequence at the N-terminus immediately after the start ATG. Repair templates for the GFP strains were generated by PCR amplifying Superfolder GFP from pDONR221. All the strains generated in this study can be found in Supplementary Table S2. The list of crRNAs (Horizon Discovery Ltd.) and primers (IDT Inc. or Eurofins genomics) used for generating repair templates and for PCR screening to confirm successful edits are listed in Supplementary Tables S3, S4 respectively.

The C-terminal OLLAS-tag for RPN-6.1 was generated by inserting the 42 bp OLLAS sequence, 5′-tcc​gga​ttc​gcc​aac​gag​ctc​gga​cca​cgt​ctc​atg​gga​aag-3′ immediately before the stop codon (TGA) in *rpn-6.1*. An ssODN was used as the repair template and contained a minimum of 35 bp homology arms to the genomic region 5′ of the insertion site, the 42 bp OLLAS sequence, and then a minimum of 35 bp homology arms to the genomic region 3′ of the insertion site (Supplementary Table S4). Appropriate silent mutations were included in the ssODN to prevent recutting of the edited sequence by the crRNA. As the OLLAS sequence contains a SacI restriction enzyme site, PCR screening to confirm *rpn-6.1::OLLAS* edits was followed by SacI restriction enzyme digest and agarose gel electrophoresis.

### RNA interference treatment

**FIGURE 2:**
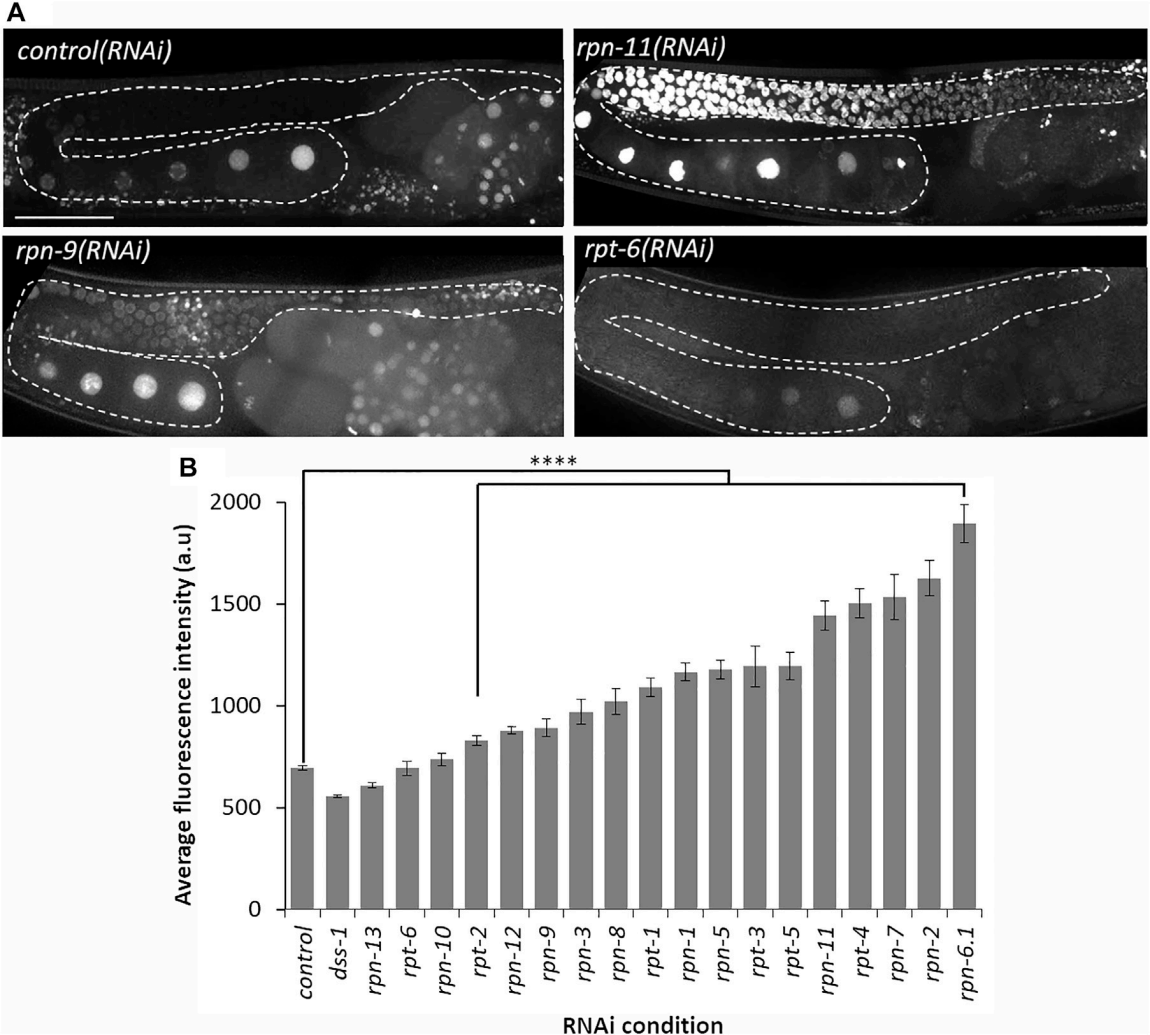
Depletion of most 19S RP subunits severely decreases proteolytic activity. **(A)** Representative images of germ line from Ub(G76V)::GFP::H2B animals treated with the indicated RNAi. Representative images of normal germline proteolytic activity [*control(RNAi)* and *rpt-6(RNAi)*], severe dysfunction of proteolytic activity [*rpn-11(RNAi)*], and moderate dysfunction of proteolytic activity [*rpn-9(RNAi)*]. A gonad arm is outlined with white dashed lines. **(B)** Average fluorescence intensity of Ub(G76V)::GFP::H2B germ lines treated with either RNAi against a control (*n* = 122) or any of the various 19 subunits of the 19S RP (*n* = 10–52). Fluorescence intensity (a. u) was measured in the region outlined with the white dashed lines as indicated in **(A)**. All images taken at the same laser intensity and PMT gain, and then the same post-image modifications made to each image. **** represents *p*-values < 0.0001 compared to *control(RNAi)* condition. Error bars represent SEM. Scale bar, 50 µm.

**FIGURE 3:**
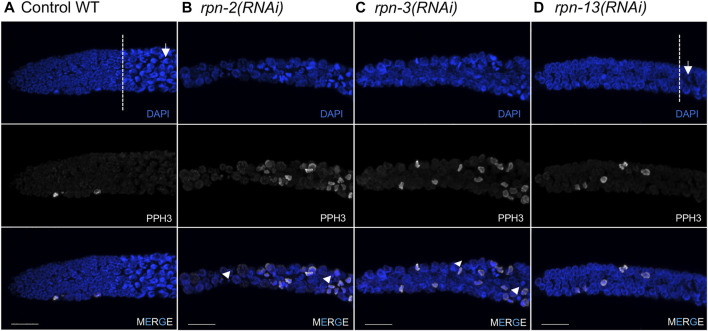
Defects in the mitotic germ line result from 19S RP subunit knockdown. Representative images of the distal tip of the *C. elegans* germ line visualized with DAPI (blue) and phospho-H3 Ser10 (white). **(A)** Wild type N2 controls [white dash line indicates start of transition zone with characteristic crescent shape nuclei (white arrow)]. **(B,C)** Worms treated with *rpn-2(RNAi)* or *rpn-3(RNAi)* presented an increased number of cells in M phase and the presence of small or fragmented nuclei (white arrowheads). Both also had shorter mitotic tips with no clear transition zone. **(D)** *rpn-13(RNAi)* resulted in no cell cycle defects, presenting mitotic tips comparable to WT worms and obvious transition zone (white dashed line) and TZ nuclei (white arrow). Images show max projections of Z stacks halfway through each gonad. Distal is to the left in all images. Scale bar, 10 μm.

**FIGURE 4:**
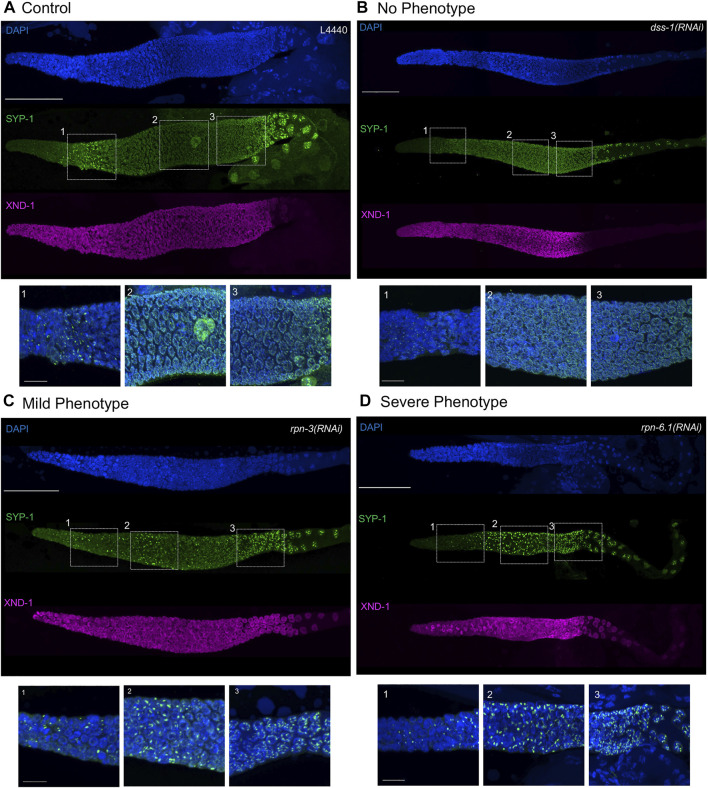
Synaptonemal complex defects are observed upon knockdown of 19S proteasome subunits. Representative images of germ lines visualized with anti-SYP-1 to mark the synaptonemal complex (green), anti-XND-1 (purple), and DAPI to mark DNA (blue). **(A)** Control, empty vector, shows the expected formation of a few SC polycomplexes (PCs) in the TZ. **(B)** No phenotype: full polymerization of SYP-1 throughout pachytene stage and correct timing of polarization to the short arm of the chromosome at diplotene comparable to control. **(C)** Mild-phenotype: extended region of PCs reaching early pachytene, with an abundant number of nuclei with fully polymerized SC in mid-pachytene. Premature polarization is also observed. **(D)** Severe phenotype: extended region of PCs into mid-pachytene, with almost all nuclei having at least one PC and no polymerization of SYP-1. Premature polarization of SYP-1 was present at late pachytene. Whole gonad scale bar, 50 m. Zoom in boxes correspond to: (1) Transition Zone, (2) Early-Mid Pachytene, (3) Late Pachytene. Scale bar, 10 m.

**FIGURE 5:**
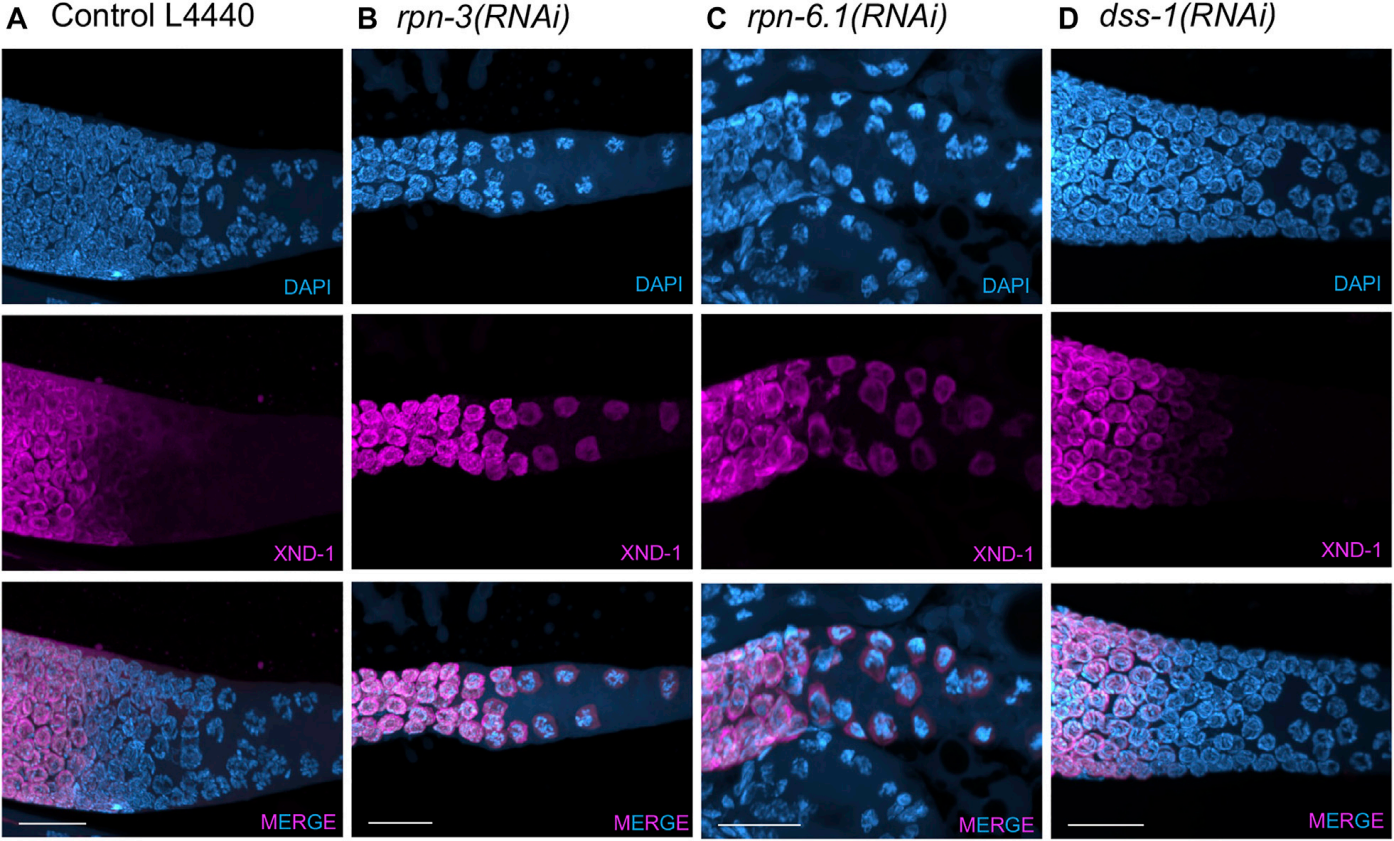
XND-1 turnover is affected by knockdown of a subset of 19S RP non-ATPase subunits. Representative images showing defects in XND-1 turnover after depletion of a specific group of non-ATPase proteasome subunits. Anti-XND-1 (magenta); DAPI stained DNA (cyan). **(A)** Vector control. **(B)** *rpn-3(RNAi)*, and **(C)** *rpn-6.1(RNAi)* are examples of two subunits whose knockdown causes persistence of high levels of nucleoplasmic XND-1 in late pachytene nuclei. **(D)** *dss-1(RNAi)* is representative of the class of subunits who depletion does not affect XND-1. Scale bar, 50 µm.

**FIGURE 6:**
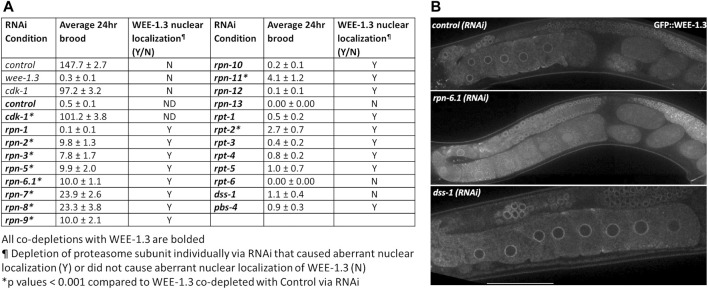
WEE-1.3 function and localization are altered by depletion of specific proteasome subunits. **(A)** Average 24 h brood and WEE-1.3 nuclear localization status of hermaphrodites treated with either *control(RNAi), wee-1.3(RNAi), cdk-1(RNAi)* individually or co-depleted with WEE-1.3, or 19S RP subunits co-depleted with WEE-1.3 *via* RNAi (all co-depletions with WEE-1.3 are bolded). All co-depletion conditions were compared to WEE-1.3 co-depleted with the control RNAi condition. * represents *p* values < 0.001, Y (yes) or N (No) represents whether or not aberrant nuclear localization of WEE-1.3 occur when control or proteasome subunits depleted individually. ND indicated not determined. **(B)** Live imaging of gonads from strain WDC2 *wee-1.3[ana2(gfp::wee-1.3)]* treated with either *control(RNAi), rpn-6.1(RNAi)* or *dss-1(RNAi)*. All images were taken at the same laser intensity and PMT gain. Scale bar, 100 µm.

RNAi treatments were done *via* RNAi feeding as previously described (Timmons et al., 2001; Allen et al., 2014; Boateng et al., 2017). RNAi clones were obtained from either the Ahringer RNAi library (*rpn-1, rpn-10, rpn-13, dss-1, rpt-1, rpt-3, rpt-6, pbs-2,* and *pbs-4*) or Open Biosystems ORF-RNAi library (Huntsville, AL) (*smd-1, wee-1.3, cdk-1, rpn-2, rpn-3, rpn-6.1, rpn-7, rpn-9, rpn-11, rpn-12, rpt-2, rpt-4,* and *rpt-5*). RNAi clones for *rpn-8* and *rpn-*5 were generated in the lab (see below for details). All RNAi clones were freshly transformed into *E. coli* strain HT115 cells before usage. Either the L4440 empty vector or *smd-1 (RNAi)* were used as a control RNAi condition for all RNAi treatments. *smd-1 (RNAi)* was utilized because it activates the RNAi response yet has no reported reproductive phenotype in a wild-type genetic background. RNAi co-depletions were performed by measuring the optical density at 600 nm wavelength of the RNAi overnight culture for each construct and then mixing the cultures in 1:1 ratio. We performed RNAi knockdown of the genes of interest by feeding the worms for a total of either 24 h at 24°C starting from L4 stage (Figures 1, 2, 6, 8 and Supplementary Figures S1, S2, S8) or 48 h, from larval stage 4 (L4) to day 2 adult at 20°C (Figures 3–5; Supplementary Figures S3–S5) as indicated.

### RNAi clone generation

RNAi feeding clones for *rpn-5* and *rpn-8* were generated by TA cloning a PCR product containing a genomic sequence of the appropriate gene into the MCS of pL4440 RNAi feeding vector. To generate clones, a 1143 bp region of *rpn-5* and 504 bp region of *rpn-8* was PCR amplified using MyTaq™ DNA Polymerase (Bioline Cat. No. 21105). The following primers were used: for *rpn-5,* forward oAKA277 5′-aat​ggc​tat​cgc​aaa​gat​gg-3′ and oAKA278 reverse 5′-gtc​agt​ttg​tgc​acg​ttg​ct-3’; and for *rpn-8,* forward oAKA392 5′-gcg​ttt​ctc​act​gtt​atg​tcg -3′ and reverse oAKA393 5′-cca​tgt​cga​gga​acc​atg​ta-3’. In brief, the vector was linearized with EcoRV, gel-extracted (Bioline Cat. No. BIO-52059), T-tailed, desalted with a DNA Clean Concentrator kit (Zymo Research Cat. No. D4004), and then ligated with either of the previously mentioned PCR product using Quick-Stick ligase (Bioline Cat. No. BIO-27027). Newly generated RNAi clones were transformed into HT115 cells and sequenced using the M13 forward universal primer to confirm successful cloning (Eurofins Genomics).

### Fertility assays

24-h total brood assays on RNAi-treated worms were performed using the previously published protocol with a minimum of three independent trials (Boateng et al., 2017). Statistical analyses were performed in Microsoft Excel using the Student *T*-test to find significant differences between the average 24-h brood of control and experimental RNAi conditions. Standard error of the mean (SEM) was calculated by dividing the standard deviation by the square root of the sample size.

### Live imaging

All fluorescent strains were treated with appropriate RNAi condition at 24°C for 24 h before imaging. 10 µl of anesthetic (0.1% tricane and 0.01% tetramisole in 1X M9 buffer) was added to a 3% agar pad on a slide and 10–15 live worms were transferred to the drop of anesthetic. A glass coverslip was slowly lowered to cover the samples and the coverslip edges were sealed with nail polish and allowed to dry before imaging. Images were obtained on a Nikon Ti-E-PFS inverted spinning-disk confocal microscope using a 60X 1.4NA Plan Apo Lambda objective. The microscope consists of a Yokowaga CSU-X1 spinning disk unit, a self-contained 4-line laser module (excitation at 405, 488, 561, and 640 nm), and an Andor iXon 897 EMCDD camera. Fluorescence intensities were quantified and image editing done using NIS-elements software.

### Immunofluorescence of proximal germ line

The tube staining method was performed on dissected gonads fixed in 3% paraformaldehyde and methanol (Chen and Arur, 2017). The samples are washed using 1X PBST (0.1% tween), blocked with 30% NGS and incubated with primary antibodies at 4°C overnight. Appropriate secondary antibodies were added and incubated at room temperature for 1–2 h followed by three washes with 1X PBST with DAPI included in the final wash and samples were mounted on a 3% agar pad with Vectashield mounting medium. The primary antibodies used in this study are: Rat monoclonal OLLAS epitope tag antibody (1:200, Novus Biologicals, Cat. No. NBP1-06713) and Rabbit anti-phospho-Histone H3 (Ser10) antibody (1:200, EMD Millipore Cat. No. 06–570). Secondary antibodies were goat-anti-rat Alexa Fluor 568 nm and goat-anti-rabbit Alexa Fluor 488 (1:1,000, Invitrogen). pH3 was used as a control of the staining protocol allowing us to identify mature oocytes.

### Immunofluorescence of synapsis phenotypes in distal germ line

For the study of synapsis, germ lines from N2, *rrf-1* or *ppw-1* worms exposed to 48 h RNAi by feeding, were dissected in 1X Sperm Salt Buffer (50 mM PIPES pH 7.0, 25 mM KCl, 1 mM MgSO_4_, 45 mM NaCl, 2 mM CaCl_2_), followed by permeabilization with 2% Triton and then fixed in the same buffer containing 2% paraformaldehyde for 5 min. Slides were placed on a frosted metal plate on dry ice before removing the coverslip and then placed in 4°C absolute ethanol for 1 min. Slides were then washed three times for 10 min each in PBST (1x PBS, 0.1% Tween) plus 0.1% BSA and incubated overnight at 4°C with the primary antibodies diluted in PBST. Following three washes of 10 min each in PBST plus 0.1% BSA, slides were incubated in the dark at room temperature for 2 h with secondary antibodies diluted in PBST. Following three 10 min washes with PBST, slides were counterstained with DAPI in the second wash and mounted using Prolong Gold antifade reagent with DAPI (Invitrogen). The primary antibodies used in this study are: Chicken anti-SYP-1 (1:1,000, courtesy of Dr. Enrique Martinez-Perez) (Silva et al., 2014); Guinea Pig anti-XND-1 (1:2,000) (Wagner et al., 2010); Rabbit anti-Histone H3 phospho Ser 10 (1:1,000) (Cell Signaling, Danvers, MA) and anti-HTP-3 (1:2,000) (Das et al., 2022). XND-1, a chromatin factor responsible for the global distribution of crossovers in *C. elegans*, was used as a control of the staining protocol allowing us also to identify the late pachytene stage in the germ line. Secondary antibodies were goat-anti-chicken Alexa Fluor 488nm, goat-anti-guinea pig Alexa Fluor 633 nm, and got anti-rabbit Alexa Fluor 568 (all diluted 1:2,000, Invitrogen).

## Results

### Differential roles of 19S RP subunits in reproduction and larval growth

We wanted to determine the effects on *C. elegans* hermaphrodite fertility upon downregulation of individual 19S RP lid and base subunits in comparison to global proteolytic inhibition via the chemical proteasome inhibitor bortezomib. As observed previously, general inhibition of the proteasome with bortezomib resulted in animals with severely reduced fertility, less then 10 progeny in a 24 h period (Table 2) (Fernando et al., 2020). RNAi knockdown of proteasome subunits also led to significant brood size reductions compared to control RNAi (Figure 1C, *p* value <0.01). Whereas the majority of 19S base subunit-knockdown animals had fewer than six offspring (<0.4% of control), *rpt-6(RNAi)* and *rpn-13(RNAi)* animals produced substantial numbers of eggs (∼25 and ∼63% of controls, Figure 1C) many of which hatched (Figure 1D). By contrast, knockdown of only half of the proteasome lid subunits severely reduced broods (<10 eggs); the remainder gave brood sizes 30%–80% the size of controls (Figure 1C). Of those with substantial numbers of eggs, *rpn-5* showed strong embryonic lethality, resulting in few to no viable offspring (Figure 1D). Importantly, our hatching results replicate the findings of Takahashi et al. (2002) where a number of the proteasome subunits were examined upon RNAi depletion for embryonic and post-embryonic lethality defects.

In the RNAi depletion studies, we cannot distinguish whether the embryonic lethality results from maternal deficits in oocyte development, from loss of proteasome function in the developing embryo due to persistence of the dsRNA, or both. Since many of the phenotypes we see in the germ line require prolonged RNAi exposure (>24 h) to be manifest (see below), at least some of the phenotypes likely reflect embryonic requirements, consistent with the zygotic requirement for proteasome function (Takahashi et al., 2002). We note that many proteins required for embryonic viability were identified in a genome-wide analysis of ubiquitinated proteins. These include multiple ribosomal subunits, the polyadenylation enzymes PAB-1 and PAB-2, and vitellogenins, among others (Koyuncu et al., 2021). In the maternal germ line, defects in germ cell proliferation, SC assembly and redistribution, and WEE-1.3 localization (discussed below) could all result in defective oocytes that would not support embryonic viability. In some instances, such as *rpt-6(RNAi)* and *rpn-9(RNAi)*, the hatched embryos developed into larvae but exhibited severe developmental defects, such as L1-L2 developmental arrest and a protruding vulva phenotype, respectively (data not shown). This data, combined with previously published data, suggests that while most of the lid and base subunits of 19S RP of the 26S proteasome play essential roles during *C. elegans* hermaphrodite reproduction, individual 19S RP subunits may play differential roles in this process.

### Downregulation of most, but not all, 19S RP subunits causes dysfunction of the proteolytic activity of the proteasome

*In vivo* fluorescent reporter systems have been developed to qualitatively assess the proteolytic activity of the 26S proteasome in whole animals and in specific tissues under various conditions (Pispa, Matilainen and Holmberg, 2020). This technique takes advantage of a translational fusion of a mutated, non-hydrolysable ubiquitin moiety to a fluorescent reporter, thereby subjecting the fluorescent protein to continuous proteasomal degradation (Dantuma et al., 2000; Hamer, Matilainen and Holmberg, 2010; Liu et al., 2012). Here, we use the published IT1187 strain with a mutated ubiquitin fused to a GFP-tagged histone protein and driven by a germline specific promoter (*pie-1*_*pro*_::Ub(G76V)::GFP::H2B::*drp-1* 3′UTR) (Kumar and Subramaniam, 2018). GFP can thus be used as an indicator of germline proteolytic activity upon RNAi depletion of specific 19S RP subunits (Fernando, Elliot and Allen, 2020). If the proteolytic activity of the proteasome is normal, the non-hydrolysable mutated ubiquitin will target the GFP::H2B for continuous proteasomal degradation leading to dim or no GFP signal in the hermaphrodite germ line. Dysfunction of the proteolytic activity of the 26S proteasome with the chemical bortezomib was previously shown to lead to accumulation of Ub(G76V)::GFP::H2B resulting in bright GFP throughout the germ lines (Fernando, Elliot and Allen, 2020).

RNAi depletion of all of the lid subunits except *rpn-10*, *rpn-13*, *dss-1/rpn-15*, and *rpt-6* resulted in bright, nuclear, germline fluorescence of the Ub(G76V)::GFP reporter compared to control RNAi-treated germ lines (Figure 2A; Supplementary Figure S1). To compare proteolytic activity of these components, we quantified the GFP intensity in germ lines depleted of specific 19S RP subunits and imaged them under the same microscopy conditions (Figure 2B). This confirmed our qualitative observations that RNAi depletion of lid subunits does not uniformly impact germline proteolytic activity. For example, depletion of *rpt-2*, *rpn-9* or *rpn-12* resulted in only a modest increase in GFP fluorescence whereas RNAi of *rpn-2, rpn-7,* and *rpn-6.1* exhibited the greatest increase in fluorescence (Figure 2B). One trivial explanation for these differences in fluorescence and phenotypes are differential sensitivity of the proteasome genes to RNAi perturbation. We do not favor this explanation at least for *rpn-9* and *rpn-12*: our fluorescent reporters (described below) allowed us to ascertain that subunit expression can be effectively inhibited even for those subunits where we observe little to no phenotypic changes (Supplementary Figure S2). Therefore, we speculate that specific 19S RP proteasome subunits may contribute uniquely to the proteolytic activity in the germ line.

### Downregulation of specific 19S RP subunits causes cell cycle defects in the adult germ line

The ubiquitin proteasome system plays a central role in cell cycle regulation [reviewed in (Zou and Lin, 2021)]. In the *C. elegans* germ line, the mitotic cells reside in the distal tip, or proliferative zone (PZ), and provide the pool of cells that enter meiosis as they move proximally (Figure 1B). Under normal growth conditions on day one of adulthood, ∼2.5% of cells have been reported to be in M phase based on staining with phospho-histone H3 (pH3) (Kocsisova et al., 2019). Accordingly, under control RNAi conditions, we observed only rare metaphase or anaphase figures in the mitotic zone and few pH3 positive cells (Figure 3A). By contrast, upon RNAi knockdown of most of the lid subunits (*rpn-3*, *rpn-5*, *rpn-6.1*, *rpn-7*, *rpn-8, rpn-9,* or *rpn-11*) and the base subunits *rpn-1* and *rpn-2*, we observed increased numbers of metaphase- or anaphase-like cells (Table 1, Supplementary Figures S3, S4) and increased numbers of pH3 positive nuclei (Figures 3B–D, Supplementary Table S5). We also observed severe defects in the PZ nuclei that are never seen in wild type: very small nuclei, fragmented nuclei, and chromosome fragments (Figures 3B,C, arrowheads). These mitotic zone defect phenotypes were also observed upon exposure to the proteasome inhibitor, bortezomib, consistent with our previous observation that inhibition of the 20S proteasome elicited cell cycle defects (Table 2) (Ahuja et al., 2017). We note that mitotic defects were also seen when E3 ligase activity was perturbed in the mitotic tip leading to aberrant activation of the ATL-1 dependent DNA damage checkpoint (Burger et al., 2013). Overall, these RNAi and drug exposures led to shorter PZs with heterodisperse nuclear sizes and shapes compared to the orderly and uniform mitotic regions of controls. These phenotypes were also accompanied by a change in nuclear morphology at meiotic entry. In wild-type and control RNAi-exposed animals, the transition zone (TZ) nuclei (corresponding to leptotene/zygotene stages of meiosis) have a distinctive crescent shape (Hillers et al., 2017). After 48 h of exposure to proteasome RNAi, the TZ nuclei were difficult to distinguish from the anaphase-like chromosomes in the mitotic region (Crittenden et al., 2006; Hubbard, 2007) (Figure 3). In contrast to the profound proliferative defects described above, RNAi knockdown of the non-ATPase subunits *rpn-10*, *rpn-12*, *rpn-13,* and *dss-1/rpn-15* did not alter PZ nuclear size or morphology and they appeared indistinguishable from control worms in this region (Supplementary Figure S5 and data not shown).

**TABLE 1:** Percentage of worms that presented cell cycle defects after knocking down proteasome non-ATPase subunits.

Gene RNAi (n)	Normal PZ (%)	Abnormal mitotic tip	
		↑M phase nuclei	Small or fragmented nuclei
*rpn-1* (10)		80%	100%
*rpn-2* (10)	10	90%	70%
*rpn-3* (7)		100%	100%
*rpn-5* (9)		78%	100%
*rpn-6.1* (11)	9	91%	82%
*rpn-7* (10)		90%	100%
*rpn-8* (10)		100%	90%
*rpn-9* (9)	44	56%	56%
*rpn-10* (6)	100		
*rpn-11* (8)		88%	100%
*rpn-12* (10)	100		
*rpn-13* (11)	100		
*dss-1* (9)	100		
*N2* WT (10)	100		

**TABLE 2:** Summary of the germline phenotypes associated with RNAi-depletion of the various 19S RP subunits.

Gene RNAi	Emb lethal^a^	Effect on brood^b^	Effect proteolytic activity^c^	MZ defects^d^	PCs/Premature polarization^e^	Defective XND-1 turnover^f^	Aberrant nuclear WEE-1.3^g^	Suppress *wee-1.3(RNAi)* infertility^h^
*rpn-1*	1	1	+	+	+/+	+	+	no
*rpn-2*	1	1	+	+	+/+	+	+	+
*rpn-3*	1	1	+	+	+/+	+	+	+
*rpn-5*	1	2	+	+	+/+	+	+	+
*rpn-6.1*	1	1	+	+	+/+	+	+	+
*rpn-7*	1	1	+	+	+/+	+	+	+
*rpn-8*	1	1	+	+	+/+	+	+	+
*rpn-9*	3	3	+	+	no	no	+	+
*rpn-10*	5	2	no	none	no	no	no	no
*rpn-11*	1	1	+	+	+/+	+	+	+
*rpn-12*	4	2	+	none	no	no	+	no
*rpn-13*	5	3	no	none	no	no	no	no
*dss-1*	4	3	no	none	no	no	no	no
*rpt-1*	1	1	+	+	+/n.d	+	+	no
*rpt-2*	1	1	+	+	+/+	+	+	+
*rpt-3*	1	1	+	+	+/+	+	+	no
*rpt-4*	2	1	+	+	+/+	+	+	no
*rpt-5*	1	1	+	+	+/+	+	+	no
*rpt-6*	3	2	no	+	no	no	no	no
Control	5	4	no	none	no	no	no	no
Bortezomib	1	1	+	+	+/no	no	no	no

a 1 < 5% hatching; 2 = 5%–39%; 3 = 40%–74%; 4 = 75%–97%; 5 = no defect.

b Average 24 h brood: 1 < 10 progeny; 2 = 11–75; 3 = 76–150; 4 > 150.

c No = does not result in statistically significant difference in expression of germline proteolytic reporter. + results in a statistically significant increase in expression of the germline proteolytic reporter.

d (+) Cell cycle defects in the adult germ line after knocking down RP subunits by RNAi. None = no cell cycle defects in the adult germ line after knocking down RP subunits by RNAi.

e (+) SC polycomplexes and premature polarization of SYP-1 after knocking down RP subunits by RNAi. No = no SC polycomplexes and premature polarization of SYP-1 after knocking down RP subunits by RNAi.

f (+) Defective XND-1 turnover in late pachytene after knocking down RP subunits by RNAi. No = normal XND-1 turnover in late pachytene after knocking down RP subunits by RNAi.

g No = no WEE-1.3 nuclear localization. + results in aberrant WEE-1.3 nuclear localization.

h No = does not result in a statistically significant suppression of *wee-1.3(RNAi)* infertility. + results in a statistically significant suppression of *wee-1.3(RNAi)* infertility.

### Downregulation of specific 19S RP subunits compromises both synaptonemal complex assembly and synaptonemal complex reorganization in late pachytene

The SC is a proteinaceous, ladder-like structure that forms between homologous chromosomes and facilitates conversion of meiotic double-strand breaks into crossovers. The SC is comprised of axial elements along the length of each homolog pair and the central elements connecting them. Of the axial element proteins, HTP-3, serves the pivotal function. The central elements are comprised of six SYP proteins, whose localization and function are interdependent [reviewed in (Hillers et al., 2017)]. In TZ nuclei, the SC central region proteins self-aggregate forming polycomplexes (PCs) (Goldstein, 1986). These PCs can be seen as bright foci using immunofluorescence or live imaging of fluorescently-tagged SC proteins (Figure 4) (Rog et al., 2017). In wild type, PCs can be seen only in ∼one to four nuclei because they disappear as the SC proteins polymerize along chromosomes to form the SC (Figure 4A) (Rog et al., 2017) The PC region is extended if the SC cannot polymerize, for example due to defects in SC regulatory proteins, among others (Couteau and Zetka, 2005; Martinez-Perez and Villeneuve, 2005). Previous work from our group and others has shown that a structurally compromised proteasome core complex results in severe defects in synaptonemal complex (SC) assembly (Ahuja et al., 2017; Prasada Rao et al., 2017; Kumar and Subramaniam, 2018). Based on these results, we wanted to interrogate how these events are affected when the 19S RP subunits are knocked down. Similar to what we observed with knockdown of the 20S subunit, RNAi knockdown of *rpn-1*, *rpn-2*, *rpn-3*, *rpn-5*, *rpn-6.1*, *rpn-7*, *rpn-8, rpn-11,* or each of the rpt’s *(rpt-1–rpt-5)* resulted in an extended region of SYP-1 PCs (Figures 4C,D, Supplementary Figures S3, S4) (Ahuja et al., 2017). As shown in Figure 4, both the number of nuclei that have PCs and the size of the PCs was increased in knockdown animals after 48 h of proteasome RNAi compared to control RNAi (Figures 4C,D). In the nuclei where PC persist, little to no SC is seen on chromosomes. In the most severe germ lines, PCs can be seen into mid-pachytene, well into the region that would normally be fully synapsed (compare Figure 4D vs. Figure 4A). This is similar to what is seen after exposure to bortezomib (Table 2). In contrast to the robust phenotypes described above, the knockdown of the remainder of the non-ATPase subunits (*rpn-9*, *rpn-10*, *rpn-12*, *rpn-13* or *dss-1*) or the base subunit *rpt-6* had no obvious effect on SC assembly or on PC size, number, or persistence (Figure 4B, Supplementary Figure S5). We note that *rpn-9* is distinct in having effects on mitotic proliferation but not on PC turnover/SC assembly, raising the possibility that these processes might be differentially sensitive to loss of proteasome activity or that different subunits may substitute for *rpn-9* in some contexts.

A prior study linked mitotic defects and subsequent PC assembly to the premature accumulation of HTP-3 (Kumar and Subramaniam, 2018). The differences that we observe in *rpn-9 (RNAi)* indicate that these phenotypes are not always associated and suggests that multiple regulatory steps feeding into synapsis may be regulated by the proteasome. However, to address whether the defects we see in cell cycle and SC formation are also due to aberrant accumulation of HTP-3, we used immunohistochemistry to determine whether HTP-3 localization is affected by knockdown of the 19S subunits. We previously observed that a subset of HTP-3 can be found in SC polycomplexes upon knockdown of the 20S proteasome (Ahuja et al., 2017). As shown in Supplementary Figure S6, HTP-3 colocalizes with SC polycomplexes and the degree of defect directly correlates with the severity of the SC phenotype. Despite this severe affect on axis morphogenesis, we saw little to no misexpression of HTP-3 in mitotic nuclei, suggesting that the effect on mitotic proliferation and SC polymerization can be uncoupled.

In late pachytene, remodeling of SC occurs to facilitate bivalent formation: SYP proteins are removed from the long arm of the chromosome (relative to the crossover) and are retained and enriched on the short arm (MacQueen et al., 2002; Colaiácovo et al., 2003). The remodeling first becomes apparent in late pachytene nuclei by polarization of SC subunit into bright and dim patches seen by immunofluorescence (MacQueen et al., 2002; Colaiácovo et al., 2003). The bright patches represent the “short arms” of the chromosomes with respect to the crossover (Hillers et al., 2017). In the proteasome 20S knockdown, we observed premature polarization of SYP with patches appearing more distally than in the wild-type controls (Ahuja et al., 2017). Even upon exposure to proteasome RNAi, there are still ∼6 bright stretches per nucleus, indicating proper crossover formation. However in almost all cases, we observed a zone of intact synapsis between the early and late SC phenotypes, suggesting that the nuclei which polarized correctly executed crossover formation prior to the onset of a robust RNAi effect (Ahuja et al., 2017). The underling mechanisms leading to this phenotype is unknown. Upon 19S RP subunit RNAi, we saw complete congruence between subunits that showed early PCs and those that presented with premature polarization (Figures 4C,D, Supplementary Figures S3, S4). In the most severe RNAi exposures, the polarization began in the mid-pachytene region (Figure 4D, Supplementary Figure S3). Similarly, those genes whose knockdown did not result in accumulation of PCs also did not show the premature polarization of the SC (Figure 4B, Supplementary Figure S5).

Despite the congruence between the RNAi studies on distal SC behaviors, the inhibition of the proteasome with bortezomib did not affect SC redistribution in late pachytene. Bortezomib affects the catalytic activity of the proteasome whereas RNAi depletion of a subunit would be expected to disrupt proteasome complex formation. The lack of phenotype with bortezomib may suggest that SC polarization is affected by a non-catalytic role of the proteasome. Alternatively, it may reflect an aspect of timing: the premature polarization is seen after 48 h of RNAi exposure whereas bortezomib kills the animals after only 18 h; the affected nuclei may not move far enough proximally to see the SC polarization phenotype before the animals die from prolonged exposure to the drug.

Since bortezomib ultimately kills the animals, it is clear that proteasome inhibition can have profound effects on organismal health. We wanted to confirm that the phenotypes we observe with RNAi inhibition of the 19S subunits were not simply a consequence of a general decline in proteostasis that is making the animal sick. This is particularly critical for the early SC phenotype which could be considered analogous to age-related aggregation as seen in Alzheimer’s and related disorders. To test this hypothesis, we took advantage of two *C. elegans* strains that are defective in RNAi in different tissues: *ppw-1* in germ line tissues; *rrf-1* in somatic tissues (Sijen et al., 2001; Tijsterman et al., 2002; Kumsta and Hansen, 2012). These mutant strains therefore give phenotypes for soma-specific and germline-specific RNAi, respectively. As seen in Supplementary Figure S7, loss of proteasome function in somatic tissues had no effect on SC polymerization or polarization. This is in stark contrast to what we observe for *rrf-1* mutant animals in which severity of SC defect was indistinguishable from the effects described above for exposure of N2 animals to proteasome RNAi: mitotic zone defects, defects in polymerization of the SC, and premature polarization of the SC (Supplementary Figure S7). We also observed that *proteasome (RNAi)* on *rrf-*1, but not *ppw-1*, led to embryonic lethality (data not shown). These results strongly support the interpretation that specific proteasome targets regulate cell cycle and SC morphogenesis.

### Nuclear XND-1 levels are regulated by the proteasome

In addition to the effects previously described for proteasome inhibition in the meiotic region of the germ line, we also observed that the proteasome is required for the proper down-regulation of XND-1 (X non-disjunction factor 1) protein in late pachytene (Figure 5). XND-1 is a chromatin factor, responsible for the global distribution of meiotic crossovers in *C. elegans* (Wagner et al., 2010)*.* In wild type, XND-1 protein is localized on autosomes from the mitotic tip of the germ line until late pachytene (Wagner et al., 2010). At that time, XND-1 appears to dissociate from chromosomes and the nuclear XND-1 signal diminishes. In cellularized oocytes, prior to ovulation, the predominant pool of XND-1 protein is cytoplasmic where it remains until it is ultimately segregated into the developing germ cells of the embryo (Mainpal et al.,2015). The mechanisms by which XND-1 redistribution is regulated are currently unknown, but we hypothesized that nuclear XND-1 pools may be controlled through protein turnover. In contrast to wild-type and control RNAi-exposed animals, we observed that knockdown of *rpn-1*, *rpn-2*, *rpn-3*, *rpn-5*, *rpn-6.1*, *rpn-7*, *rpn-8* or *rpn-11*, the same subunits that altered the SC polymerization and restructuring, also led to defects in XND-1 turnover. In the late pachytene nuclei of these RNAi-exposed animals, XND-1 levels remained high and nucleoplasmic (Figure 5). Thus, we infer that these RP subunits are not required for the chromosomal association of XND-1 *per se*, but rather are responsible for the turnover and/or export of the non-chromosomally associated XND-1 pool. This phenotype of RP knockdown is particularly noteworthy because it occurs at/near the time when 1) profound changes in oocyte transcription and chromatin are occurring to prepare the oocyte for embryonic development and 2) a subset of nuclei is culled by apoptosis. Whether the proteasome plays a pivotal role(s) in promoting these transitions deserves further investigation.

### Downregulation of specific 19S RP subunits suppresses *wee-1.3(RNAi)* infertility and alters WEE-1.3 localization in oocytes

*C. elegans* oocytes, like oocytes of most sexually reproducing organisms, undergo meiotic arrest (Burrows et al., 2006; Inoue et al., 2006; Ruiz, Vilar and Nebreda, 2010). Oocyte meiotic arrest in *C. elegans* hermaphrodites is maintained by an inhibitory kinase WEE-1.3 phosphorylating the CDK-1 component of maturation promoting factor (MPF) and thus inactivating MPF (Lamitina and L’Hernault, 2002; Burrows et al., 2006; Allen, Nesmith and Golden, 2014). Depletion of WEE-1.3 in *C. elegans* causes precocious oocyte maturation resulting in infertility (Burrows et al., 2006). A large RNAi suppressor screen identified 44 suppressors that when co-depleted with WEE-1.3 suppressed the infertility defect (Allen, Nesmith and Golden, 2014). Five of the suppressor genes were subunits of the 19S RP. However not all of the 19S RP subunits were included, or identified as positives, in the aforementioned screen (Allen et al., 2014). Therefore, we systematically screened each of the 19S RP subunits to determine if there are additional subunits whose depletion suppresses *wee-1.3 (RNAi)* induced infertility.

Hermaphrodites fed *wee-1.3 (RNAi*) are infertile, averaging less than one egg per adult hermaphrodite in a 24 h period (Figure 6). In the absence of CDK-1, WEE-1.3 is dispensable. Accordingly, *cdk-1 (RNAi)* suppresses *wee-1.3 (RNAi)* infertility and therefore serves as a positive control in these studies (Figure 6A) (Burrows et al., 2006). Significant increases in brood sizes were seen when WEE-1.3 was co-depleted with 8 out of 13 of the 19S lid subunits, but only seen with co-depletion of one of the 19S base subunits, RPT-2 (Figure 6A). Depletion of the remaining five base units were unable to suppress, similar to the negative control co-depleted with WEE-1.3 (Figure 6A).

WEE-1.3 is mainly localized to the perinuclear region, but also can be seen in the cytoplasm and ER (Allen et al., 2014). Depletion of most 19S RP subunits in an endogenously GFP tagged WEE-1.3 strain [WDC2—*gfp::wee-1.3(ana2)*] caused aberrant nuclear accumulation of WEE-1.3 (Figure 6B and Supplementary Figure S8). RNAi of four of the 19S RP subunits that failed to suppress *wee-1.3 (RNAi)* sterility, RPN-10, RPN-13, DSS-1/RPN-15 and RPT-6, also showed no change in GFP::WEE-1.3 localization (Figure 6B; Table 2; Supplementary Figure S8). However, since we previously reported that *rpn-10 (ana7)*, a genetic null, results in nuclear accumulation of GFP::WEE-1.3 in oocytes, it is possible that our RNAi depletions of RPN-13, DSS-1 or RPT-6 did not give sufficient knockdown to elicit an alteration in perinuclear WEE-1.3 localization (Fernando et al., 2020). However, our previous study also reported that chemical inhibition of the proteolytic activity of the proteasome with bortezomib neither suppressed *wee-1.3 (RNAi)* infertility nor induced nuclear accumulation of WEE-1.3 (Fernando et al., 2020). Therefore, we favor the conclusion that a fully intact 19S RP is required for the proper localization of WEE-1.3 in oocytes and that this role is independent of the proteasome’s role in proteolysis.

### Ubiquitous somatic and germline expression of 19S RP lid subunits RPN-7, RPN-8, and RPN-9

**FIGURE 7:**
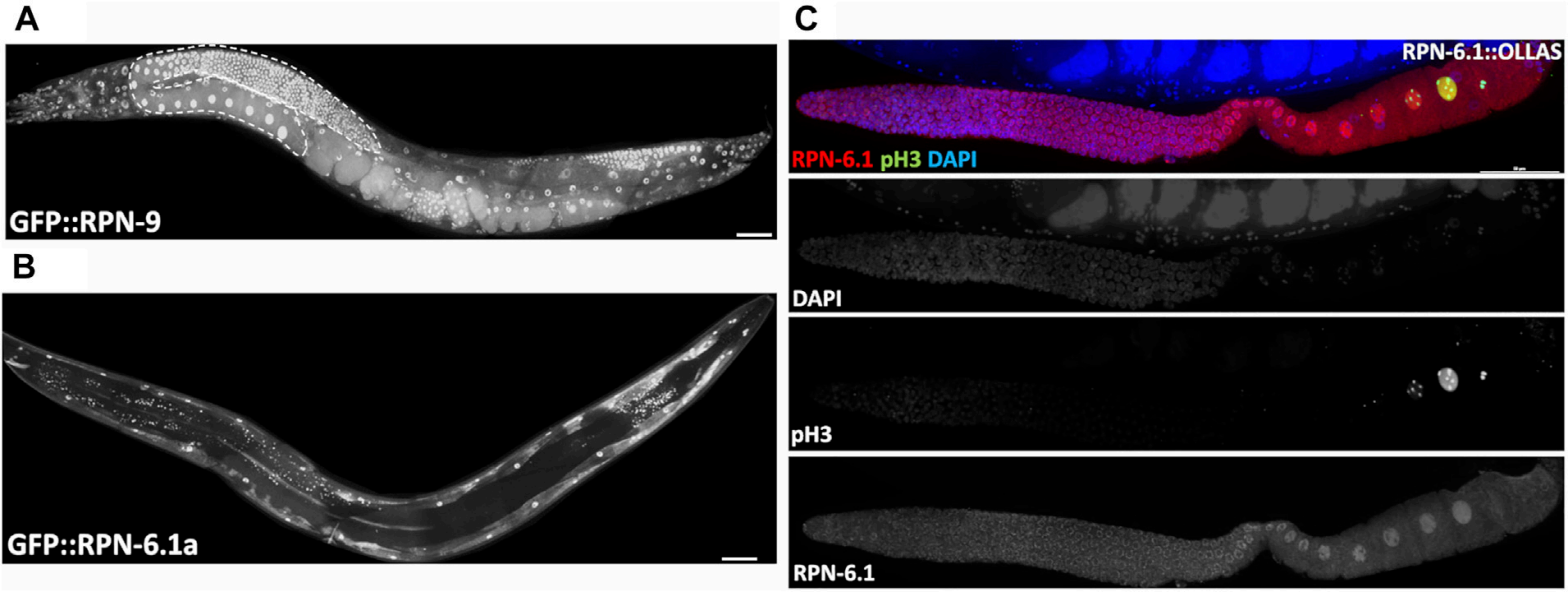
The two RPN-6.1 isoforms exhibit different spatial localization. Live imaging of hermaphrodites expressing endogenously GFP-tagged **(A)** RPN-9 and **(B)** RPN-6. Strains are WDC5 *rpn-9[ana5 (gfp::rpn-9)]* and WDC3 *rpn-6.1a [ana3(gfp::rpn-6.1a)]*. **(C)** Immunofluorescence image of *rpn-6.1[ana12(rpn-6.1::ollas)]* strain dissected gonad co-stained with anti-OLLAS (red), anti-pH3 (green, condensed chromatin) and DAPI for DNA (blue). Anti-pH3 was used solely to confirm presence of maturing oocytes. Bright nuclear and relatively dim cytoplasmic RPN-6.1b expression shown throughout germ line. Scale bar, 50 µm.

The transparency of *C. elegans* makes it an excellent model to conduct live imaging of fluorescently tagged proteins and is useful to study highly dynamic protein complexes such as the 26S proteasome. To better understand the spatiotemporal expression of 19S RP subunits *in vivo* and ultimately to perform future biochemical analyses, we set out to endogenously tag each of the 19S RP subunits with GFP or OLLAS. We previously reported that an endogenous GFP::RPN-12 strain exhibits somatic and germline expression (Fernando et al., 2020). N-terminal GFP fusions with RPN-7, RPN-8, or RPN-9 showed ubiquitous expression in both the nuclei and cytoplasm of germline and somatic cells, including developing oocytes (Figure 7A and Supplementary Figure S9). This subcellular expression matches that determined by antibody staining against subunits of the proteasome core particle in *C. elegans* and in other systems (Brooks et al., 2000; Mikkonen et al., 2017; Kumar and Subramaniam, 2018; Fernando et al., 2020). Importantly, all three of these strains exhibited no effect on lifetime brood size and only a moderate reduction in lifespan when compared to wild-type control animals (data not shown).

### Expression of the 19S RP lid subunit RPN-6.1a is restricted to the body wall muscle

While the 19S RP subunits (RPN-7, -8, -9, and -12) all exist as a single protein isoform, the RPN-6.1 subunit has two protein isoforms, A and B, that differ by an extension of the N-terminus in RPN-6.1A (Supplementary Figure S10) (*WBGene00004462, version: WS284*, 2021). A strain endogenously tagging the N-terminus of RPN-6.1A with GFP shows nuclear and cytoplasmic GFP expression restricted to the body wall muscle cells of the animal {Figure 7B, strain WDC3 *rpn-6.1a [ana3(gfp::rpn-6.1a)]}*. Since an N-terminal fusion of RPN-6.1B would impact expression of RPN-6.1A, we instead attempted to infer its expression from an endogenous GFP tag to the C-terminus of RPN-6.1, which would simultaneously tag both RPN-6.1 isoforms (Supplementary Figure S10). Unfortunately, we were unable to obtain viable or fertile RPN-6.1::GFP animals, suggesting GFP interfered with the proper folding or function of RPN-6.1. Instead, we were able to create a functional gene fusion using a small epitope tag, OLLAS {WDC12 *rpn-6.1[ana12(rpn-6.1::ollas)]*}. Lifespan and lifetime brood assays of the *gfp::rpn-6.1a* and *rpn-6.1::ollas* strains demonstrated that the N-terminal tag had no effect compared to wild-type control animals, while the C-terminal OLLAS tag results in a slightly reduced lifetime average brood and lifespan compared to wild-type control (data not shown).

We immunostained dissected RPN-6.1::OLLAS animals with an anti-OLLAS antibody and as predicted, we observed staining in the nuclei and cytoplasm of germline and intestinal cells (Figure 7C and Supplementary Figure S11). Since GFP::RPN-6.1A fluorescence was restricted to the body wall muscle, the anti-OLLAS staining that we observed in the germ line and intestine can be inferred to be due to the expression of RPN-6.1B. Interestingly, sperm did not exhibit expression of either isoform RPN-6.1A or B (data not shown). We hypothesize that this may be due to the presence of a sperm-specific ortholog of *rpn-6.1*, *rpn-6.2*, that is reported as expressed in sperm (Dr. Lynn Boyd personal communication and WormBase). Additionally, neither *gfp::rpn-6.1a* nor *rpn-6.1::ollas* animals exhibit expression in the pharynx, unlike other tagged proteasomal subunits, for example *gfp::rpn-9* (Figures 7A,B; Supplementary Figure S11). This implies that the pharynx might either have a pharyngeal-specific proteasomal subunit orthologous to RPN-6.1 or that the pharyngeal proteasome does not utilize an RPN-6.1 subunit for function.

### RPN-6.1 and RPN-7 are required for nuclear localization of the 19S RP subcomplex

**FIGURE 8:**
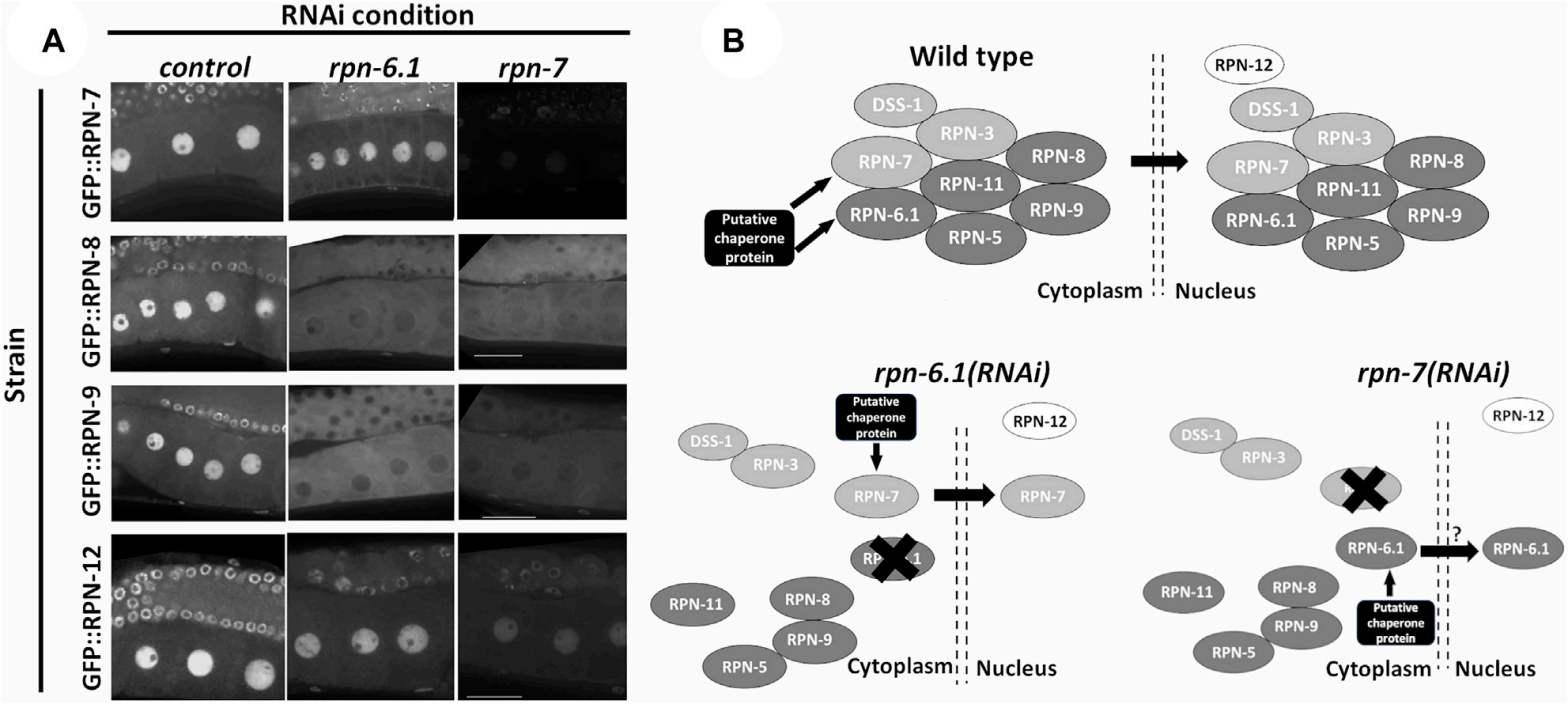
RPN-6.1 and RPN-7 are required for the nuclear localization of RPN-8 and RPN-9. **(A)** Live imaging of hermaphrodite oocytes from endogenously GFP tagged strains *rpn-7[ana1(gfp::rpn7)]*, *rpn-8[ana4(gfp::rpn-8)]*, *rpn-9[ana5(gfp::rpn-9)]* and *rpn-12[ana6(gfp::rpn-12)]* treated with either *control(RNAi), rpn-6.1(RNAi)* or *rpn-7(RNAi)* (*n* = 15–42). Scale bar represents 25 µm. **(B)** Model for role of RPN-6.1 and RPN-7 in nuclear localization of 19S RP lid combining existing information on eukaryotic proteasome assembly model (Budenholzer et al., 2017; Bai et al., 2019).

Our previous results demonstrated a nuclear pool of many 19S RP subunits. To test if any *C. elegans* 19S subunits are necessary for the nuclear localization of lid subcomplex components, we downregulated individual 19S RP lid subunits via RNAi and asked whether localization of other 19S RP subunits was affected. RNAi depletion of either RPN-6.1 or RPN-7, but not other lid subunits, impacted the nuclear signal of GFP::RPN-8 and GFP::RPN-9 in oocytes (Figures 8A,B, Supplementary Figure S2). By contrast, these depletions did not impact GFP::RPN-7 and GFP::RPN-12 localization (Figure 8A). Together our data show that RPN-6.1 and RPN-7 are required for the nuclear localization of the 19S RP lid particle subcomplexes.

## Discussion

The proteasome plays critical and essential roles throughout the *C. elegans* hermaphrodite germ line to ensure various aspects of oocyte development and ensuing embryonic viability. Our RNAi depletion studies of each of the 19S regulatory particle subunits have uncovered catalytic and structural, non-proteolytic roles for the whole proteasome, lid-specific functions, as well as evidence for moonlighting roles of specific subunits. In addition, endogenous fluorescent-labeling of specific proteasomal subunits showed cellular and subcellular localization of those subunits that has not been clearly reported by previous studies.

In this study, we provide evidence for roles of the 19S RP in both oocyte development and embryonic and larval development. Individual subunits of the 19S RP of the *C. elegans* proteasome contribute to different extents to a range of germline processes. RNAi depletion of 13 out of 19 subunits of the 19S RP (Table 2) caused very high rates of embryonic lethality in progeny of treated mothers (hatching <20%; where 12/13 were <5%). All 13 of these subunits also caused severe impairment of the proteolytic activity of the proteasome as measured with the germ line proteasome reporter, Ub(G76V)::GFP::H2B and all exhibited PZ defects, impaired SC polymerization, altered XND-1 localization, and aberrant WEE-1.3 localization. By contrast, bortezomib treatment did not affect SC polarization, XND-1 localization, and aberrant WEE-1.3 localization. Although these differences could reflect the timing or magnitude of exposure, we favor a model in which these results suggest that the PZ and SC polymerization defects and effects on embryonic viability are due to the role of specific gene targets or the result of proteostatic imbalance. In addition, the comparison between RNAi depletion and bortezomib raises the possibility that an intact, stable proteasome structure, but not its catalytic activity, is required for the proper perinuclear localization of WEE-1.3, the chromatin association of XND-1, and the correct timing of SC polarization (Figures 4–6). Bortezomib works by binding to the β5 subunit of the 20S CP and inhibiting its peptidase activity, whereas depletion of specific 19S subunits may weaken 19S RP and 20S CP interactions, destabilizing part or all of the proteasome structure or may impair 19S RP-substrate interactions (Adams et al., 1999; Bai et al., 2019; Thibaudeau and Smith, 2019). While proteolytic roles of the proteasome are well established, growing evidence supports additional roles for intact proteasome (or its subcomplexes), including in the cell cycle, transcription, and chromatin organization (Nishiyama et al., 2000; Geng, Wenzel and Tansey, 2012; Seo et al., 2017). One possibility is that the proteasome tethers WEE-1.3 to the perinuclear region, potentially even the nuclear pore complex, through protein-protein interactions (Albert et al., 2017).

Stability of the proteasome complex depends on the presence of a few essential 19S RP subunits. RPN-6.1/Rpn6/PSMD11 is one of the subunits known to play a crucial role in proteasome stability and lid subcomplex assembly (Santamaría et al., 2003; Isono et al., 2005; Bai et al., 2019). Our results suggest that *C. elegans* RPN-6.1 and RPN-7 aid in the nuclear localization of the lid subcomplex (see proposed model in Figure 8B). Our future studies will focus on determining the mechanism by which RPN-6.1 and RPN-7 aid in this process. Interestingly, neither RPN-6.1 nor RPN-7 possess canonical NLS sequences, implying either the proteins have cryptic NLSs or that additional binding partners are required for nuclear localization of the 19S RP lid subcomplexes (Figure 8B). The endogenously-tagged strains that we generated will be beneficial in both biochemical and genetic experiments to identify such sequences or chaperone binding partners. Obtaining a complete set of fluorescently tagged lid subunits will aid in further elucidating the mechanism by which the lid subcomplex assembles and becomes nuclear localized using the *C. elegans* germ line as a model system.

Interestingly, RNAi depletion of the 19S RP subunits *rpn-9* and *rpn-12* moderately impaired proteolytic activity of the proteasome without severely affecting brood sizes (∼50 and ∼66% reductions) or hatching rates (∼50 and ∼20% reductions, respectively) (Figure 2B and Supplementary Figure S1). One possible explanation is that the assays reflect differential requirements for proteasome function in different cells: Ub(G76V)::GFP expression is assayed in the meiotic germ line and developing oocytes; brood sizes reflect a combination of mitotic divisions, apoptosis, and oocyte maturation; and hatching rates reflect the impacts on the laid eggs. Consistent with this interpretation, *rpn-9 (RNAi)* but not *rpn-12 (RNAi)* exhibited mitotic zone defects which could explain the brood size defects in the former. Alternatively, there may be regional or cell type-specific differences in the RNAi efficiency for these subunits or different sensitivities of these phenotypic readouts to proteasome impairment. A final possibility, relating specifically to *rpn-12*, is the previously proposed idea that *rpn-10* and *rpn-12* are redundant and can compensate for one another during oocyte development (Takahashi et al., 2002; Shimada et al., 2006; Fernando, Elliot and Allen, 2020).

Another unanticipated observation is that RNAi directed against *dss-1*, *rpn-13*, *rpn-10*, and *rpt-6* had mild to no effect on many of the processes examined. While these results may indicate that the RNAi is inefficient at knocking down these subunits, we note that all four knockdowns did have a mild effect on brood size, producing 25%–80% of the number of eggs as wild type, strongly suggesting the RNAi is working. Additionally, although 99% of the embryos hatched upon knockdown of RPN-13, most larvae presented a ruptured vulva phenotype (data not shown). These data strongly suggest that RNAi depletion of these subunits is functional. One possible model for the lack of strong phenotype is that other proteostasis mechanisms may be upregulated when these subunits are inactivated, thereby supporting development and fertility with a partially compromised proteasome. Prior studies have revealed such cross-pathway feedback mechanisms, but whether all tissues respond similarly is not clear (Li et al., 2022).

RPN-10, RPN-13, and DSS-1 are known as ubiquitin receptors of the 26S proteasome, but there is evidence to suggest that these subunits confer substrate specificity and do not function as global receptors of polyubiquitinated proteasome substrates (Shimada et al., 2006; Paraskevopoulos et al., 2014). In mammalian cells, RPN10 can compensate for loss of RPN13, and vice versa, presumably because of their shared role in ubiquitin-binding (Hamazaki et al., 2015). It would be interesting to test whether similar compensation happens in the worm. RPN-1 is the only other 19S RP subunit thought to have ubiquitin-binding activity. Since loss of RPN-1 is much more severe, we postulate that loss of only RPN-10, RPN-13, or DSS-1 may not sufficiently impair the ability of the other subunits to feed substrates to RPN-1 for movement through the base and into the proteasome core. Additionally, as previously mentioned, there is redundancy between *rpn-10* and *rpn-12* (Takahashi et al., 2002). Structural analyses place RPN-10 at the interface of the 19S base and lid, linking RPN-1 to RPN-12 (see Figure 1A). In the absence of RPN-10, these two subunits may directly interact, as suggested by dynamic models of proteasome structure with and without substrate (Bard et al., 2018). Alternatively, however, these data may suggest that the 19S lid adopts a novel structure in the worm germ line. Existence of tissue-specific proteasomes is not unprecedented but the study of these variants is still in its infancy (Kish-Trier and Hill, 2013; Uechi et al., 2014; Gómez-H et al., 2019; Motosugi and Murata, 2019). These modified proteasomes provide a mechanism to adapt to tissue-specific needs. Determining whether the *C. elegans* 19S RP adopts a germline-specific configuration is an important avenue for future investigation.

Our studies also point to differences between the behavior of the 19S lid and base. With exception of *rpt-2*, none of 19S base subunits were able to suppress *wee-1 (RNAi)*-induced sterility, whereas many of the lid subunits did suppress. These data could be explained if the lid has independent, non-proteasomal functions or that it combines with other proteins to make an alternative regulatory particle. In favor of the former model, we previously showed that proteasome inhibition by bortezomib failed to suppress *wee-1.3 (RNAi)* infertility suggesting that the misregulation of protein turnover is not driving the oocyte maturation defect of *wee-1.3* depletion (Fernando et al., 2020). The mechanism by which the suppression of *wee-1.3 (RNAi)* infertility occurs is still unknown but future studies may offer new insights into the regulation of this highly conserved WEE-1.3/Myt1 cell cycle kinase.

Further support of non-proteolytic roles for a 19S RP subunit comes from studies on RPT-6 that demonstrated that RPT-6 plays non-proteolytic roles in transcription in both yeast and mammalian cells (Chang et al., 2001; Gonzalez et al., 2002; Lee et al., 2005; Uprety et al., 2012). In *C. elegans*, RPT-6 interacts with the transcription factor ELT-2 to regulate expression of immune response genes and this role is independent of the proteolytic activity of the proteasome (Olaitan and Aballay, 2018). Therefore, our observation that depletion of RPT-6 does not affect germline proteolytic function, but rather causes a reduced brood and larval arrest can mean two things: either RPT-6 is a developmental stage specific proteasome subunit that is essential for proteolytic function of the proteasome only during larval development; or, RPT-6 may play non-proteolytic roles in the *C. elegans* germ line because depletion of RPT-6 causes a reduced brood but overall germ line proteolytic function is not affected. While we favor, off-proteasome functions for RPT-6 in controlling oocyte quality, further studies are needed to elucidate RPT-6 function.

Endogenous GFP tagging of a number of the 19S proteasomal subunits indicated strong expression throughout the germ line of *C. elegans*, in addition to ubiquitous, somatic expression. However, we are the first to report isoform-specific localization of RPN-6.1 in *C. elegans*. With isoform RPN-6.1A being expressed only in the body wall muscles while RPN-6.1::OLLAS (which marks both Isoforms A and B) is expressed throughout the hermaphrodite female germ line but is distinctly absent from both sperm and the pharynx. Since downregulation of RPN-6.1 causes severe dysfunction of the proteolytic activity of the proteasome, we speculate that there is likely to be other RPN-6.1 variant(s) that functions in the pharynx and sperm (Vilchez and Morantte, 2012; Fernando et al., 2020). Indeed, RPN-6.2, an RPN-6.1 paralog, has recently been identified as sperm-specific (personal communication, Lynn Boyd). Sperm-specific proteasome subunits have been described in various systems and may exist to meet the massive protein turnover for the histone to protamine transition or to facilitate fertilization (Belote and Zhong, 2009; Sutovsky, 2011; Uechi et al., 2014; Zhang et al., 2019; Palacios et al., 2021). One critical remaining question is whether the different isoforms reflect tissue-specific modifications or adaptations to specific substrate in these tissues. Further analysis of these questions in the worm will enhance our knowledge of the diverse and dynamic regulation of the proteasome in different tissues.

We currently do not know the mechanism of how either proteasome dysfunction or proteasome complex instability results in many of these varied germline defects. Mining of recent data of ubiquitinated proteins during aging (Koyuncu et al., 2021) provides little insight into particular germline targets, perhaps because the approach enriches for highly expressed proteins, including ribosomal proteins, extracellular matrix and actin and tubulin binding proteins, as well as proteasome subunits themselves. The dearth of germline-specific proteins that are on the list, underscores that much work still needs to be done in this area. Finding the precise targets and the ubiquitin ligases involved is a critical future direction. In addition, greater understanding of the non-proteolytic roles will require identification of direct binding partners of proteasome subcomplexes. Further analysis of moonlight functions may lead to discovery of unexpected regulators of individual proteasome subunits.

The spatiotemporal and depletion analyses of the *C. elegans* proteasome subunits in this study revealed differential roles being played by specific subunits and provides crucial information to fill the knowledge gaps in our understanding of the 26S proteasome and its many functions. Due to the lethality of proteasome knock-down, we relied on RNAi for most of these studies. Since the effectiveness of RNAi can vary gene-to-gene, we cannot rule out that the lack of phenotypes observed with some of the subunit RNAi knockdowns resulted from an inability to fully deplete protein function. Other methods such as auxin-inducible conditional knockdown of the proteasome subunits cannot be used because it relies on the proper function of the proteasome to degrade targeted protein of interest. In the future the application of tissue-specific loss-of-function alleles and/or rescue studies may allow us to overcome this limitation of our study. Generation of the endogenously fluorescently tagged 19S RP subunits reported here, and future tagged subunits, will serve as valuable resources for future proteasome studies. Our current findings in the multicellular model *C. elegans* and the future ones that stem from this research have tremendous potential to transform the proteasome field and can be translated into better understanding human proteasome function.

## Data Availability

The raw data supporting the conclusion of this article will be made available by the authors, without undue reservation.
