# Supplementary material for: Proteasomal subunit depletions differentially affect germline integrity in C. elegans
Source: Front Cell Dev Biol. 2022 Aug 17;10:901320. doi: 10.3389/fcell.2022.901320 (PMC9428126; doi:10.3389/fcell.2022.901320)
Supplement: Supplementary file 1 [file Presentation1.pdf]

Supplemental Figure 1

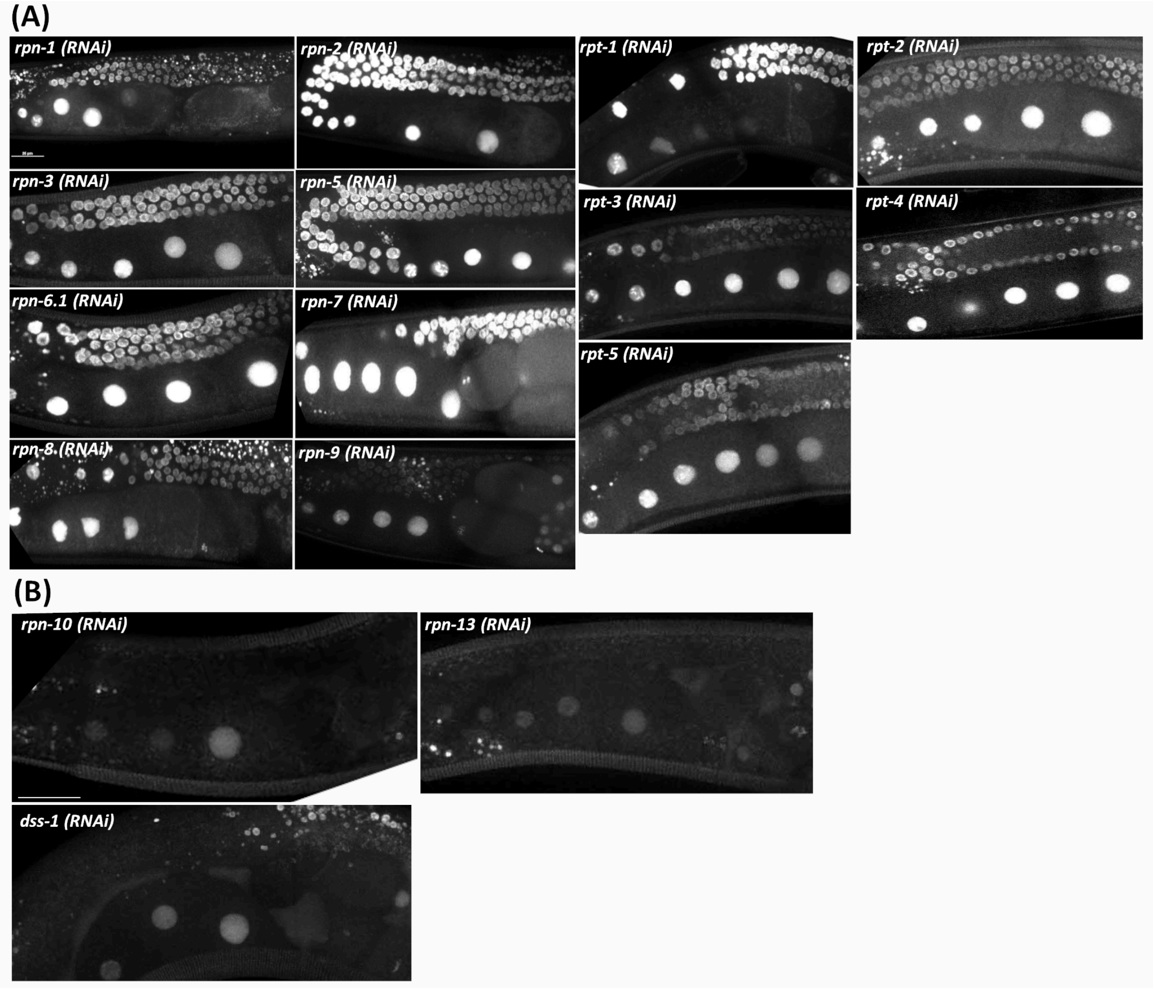

Supplemental Figure 1. Ub(G76V)::GFP::H2B animals RNA-depleted of 19S RP subunits to assess the germline proteolytic activity of the proteasome. (A) Live imaging of germ lines from Ub(G76V)::GFP::H2B animals RNAi-depleted of the indicated 19S RP subunits of the 26S proteasome. The increase in the fluorescence in Ub(G76V)::GFP::H2B germ lines indicates reduced proteolytic activity of the proteasome. (B) RNAi depletion of RPN-10, RPN-13 and DSS-1 did not increase fluorescence in Ub(G76V)::GFP::H2B germ line suggesting proper function of the proteasome.

Supplemental Figure 2

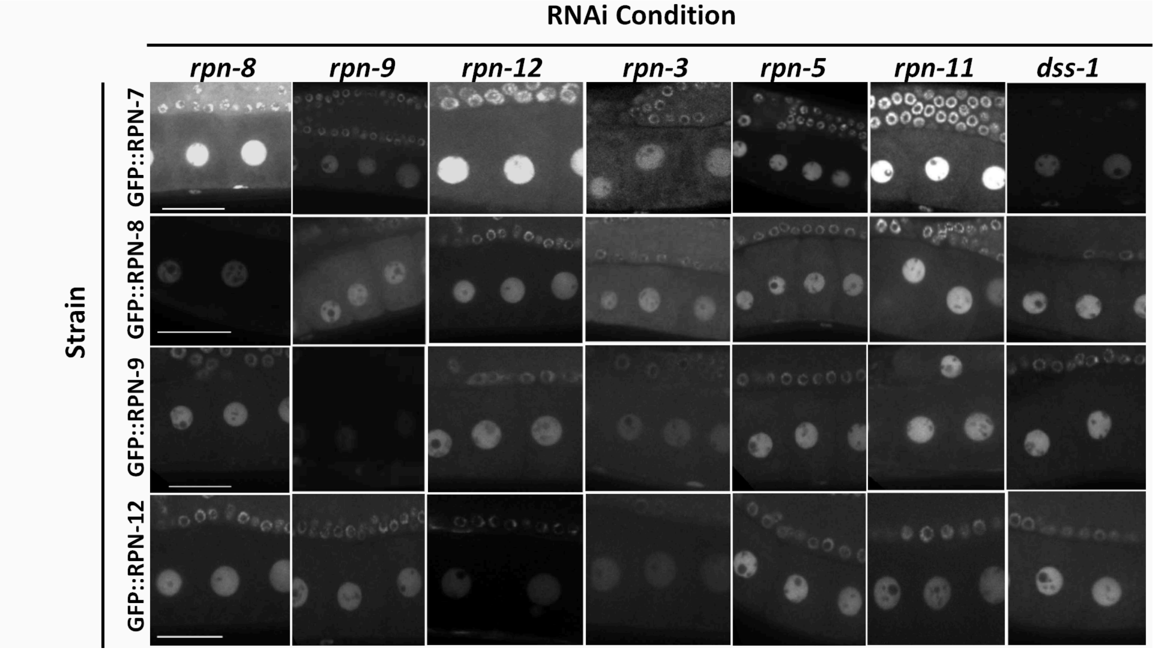

Supplemental Figure 2. Localization of specific 19S RP lid subunits under various lid subunit RNAi conditions. Depletion of RPN-8, RPN-9, RPN-12, RPN-3, RPN-5, RPN-11 and DSS-1 via RNAi in GFP::RPN-7, GFP::RPN-8, GFP::RPN-9 and GFP::RPN-12 expressing oocytes (n=15-20). Scale bar represents 25μm.

Supplemental Figure 3

A) B)

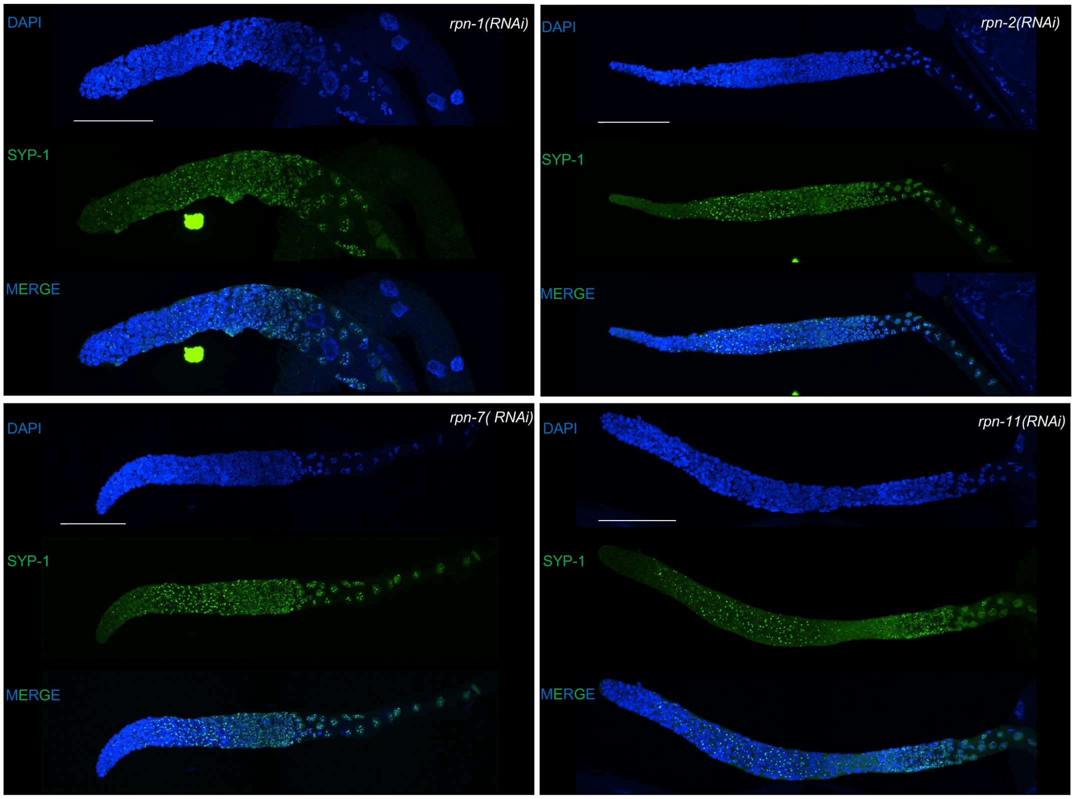

C) D)

E)

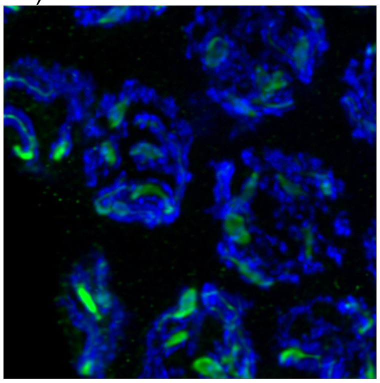

Supplemental Figure 3: RNAi knockdown of 19S RPN subunits RPN-1, RPN-2, RPN-7, or RPN-11 resulted in a severe SC phenotype. A- D) Extended region of SYP-1 PCs up until mid-pachytene, with almost all nuclei presenting at least one PC and no polymerization of SYP. Premature polarization of SYP-1 was present at late pachytene nuclei. Scale bar = 50 μm. E) Zoomed in image of nuclei showing premature polarization. The localization of the six polarized SC stretches (green) can be seen on the DNA (blue). The six stretches correspond to six short arms of the chromosome relative to the crossover site on each chromosome.

Supplemental Figure 4

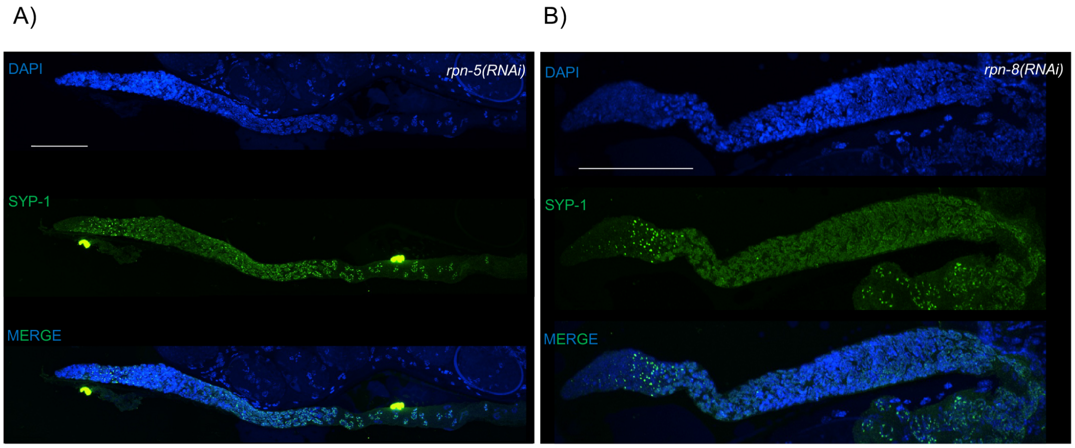

Supplemental Figure 4: RNAi knockdown of 19S RPN subunits RPN-5 or RPN-8 resulted in a mild SC phenotype. Extension of SYP-1 PCs region reaching early pachytene, with an abundant number of fully polymerize nuclei in mid-pachytene. Premature polarization is also observed at late pachytene nuclei. Scale bar = 50 μm.

Supplemental Figure 5

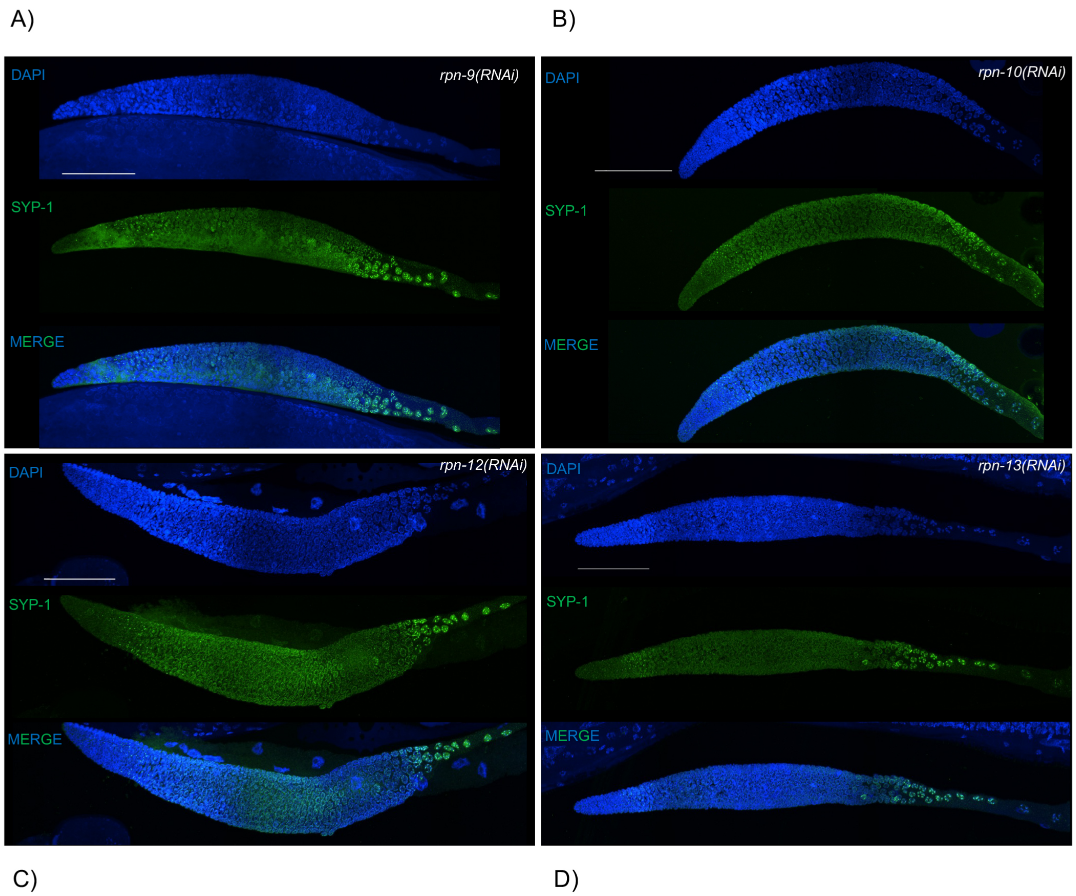

Supplemental Figure 5: RNAi knockdown of 19S RPN subunits RPN-9, RPN-10, RPN-12 or RPN-13 presented no SC phenotype. Full polymerization of SYP-1 through pachytene stage and correct timing of polarization to the short arm of the chromosome at diplotene stage comparable to control. Scale bar = 50 μm.

Supplemental Figure 6

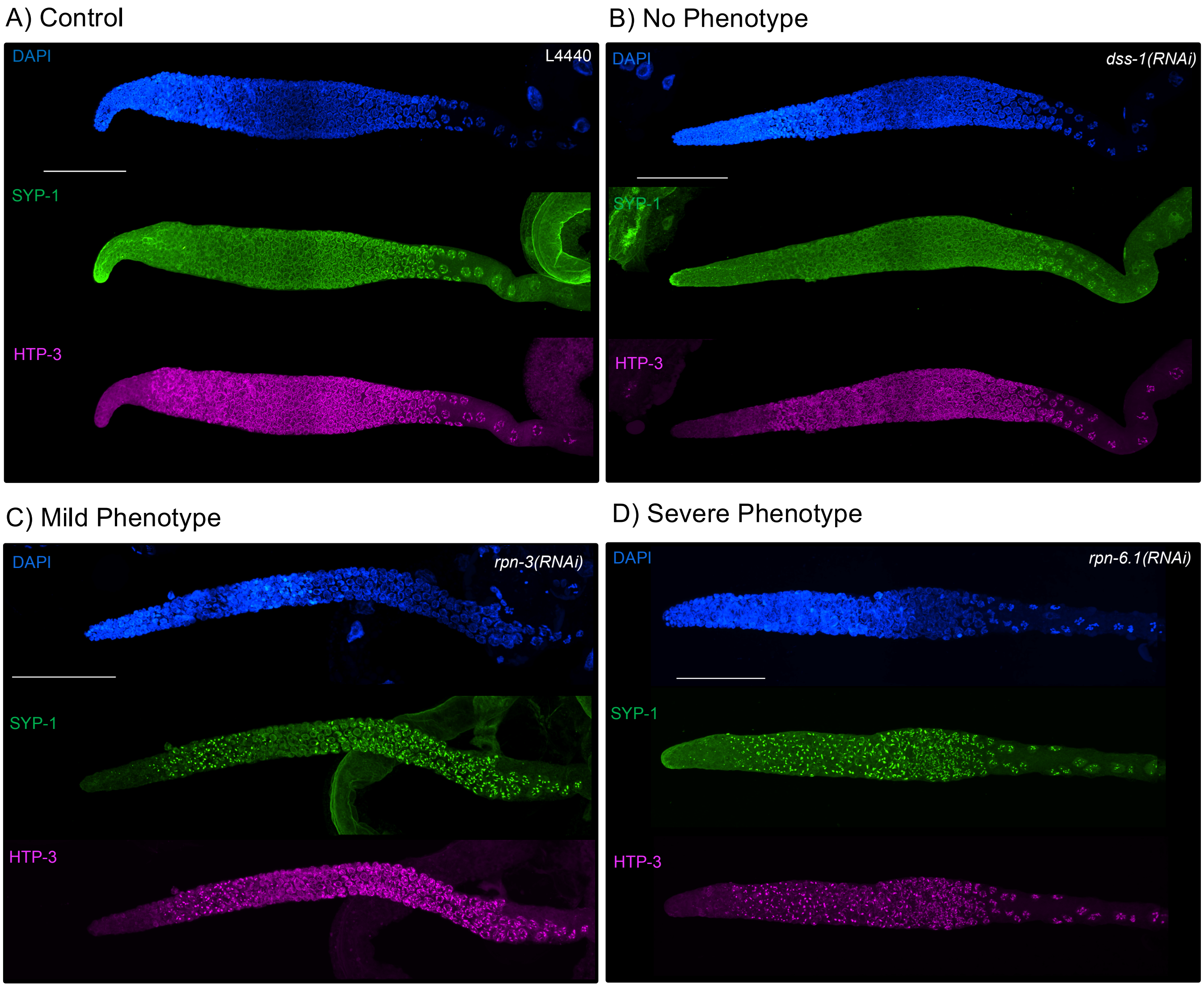

Supplemental Figure 6. HTP-3 is recruited to SC polycomplexes upon knockdown of 19S RPs. N2 worms were treated for 48 hr with proteasome 19S RP RNAi, fixed, and extruded gonads were stained with DAPI (blue), anti-SYP-1 (green) to visualize SC protein, and anti-HTP-3 (magenta) to visualize the chromosome axis. Scale bar is 50µm.

Supplemental Figure 7

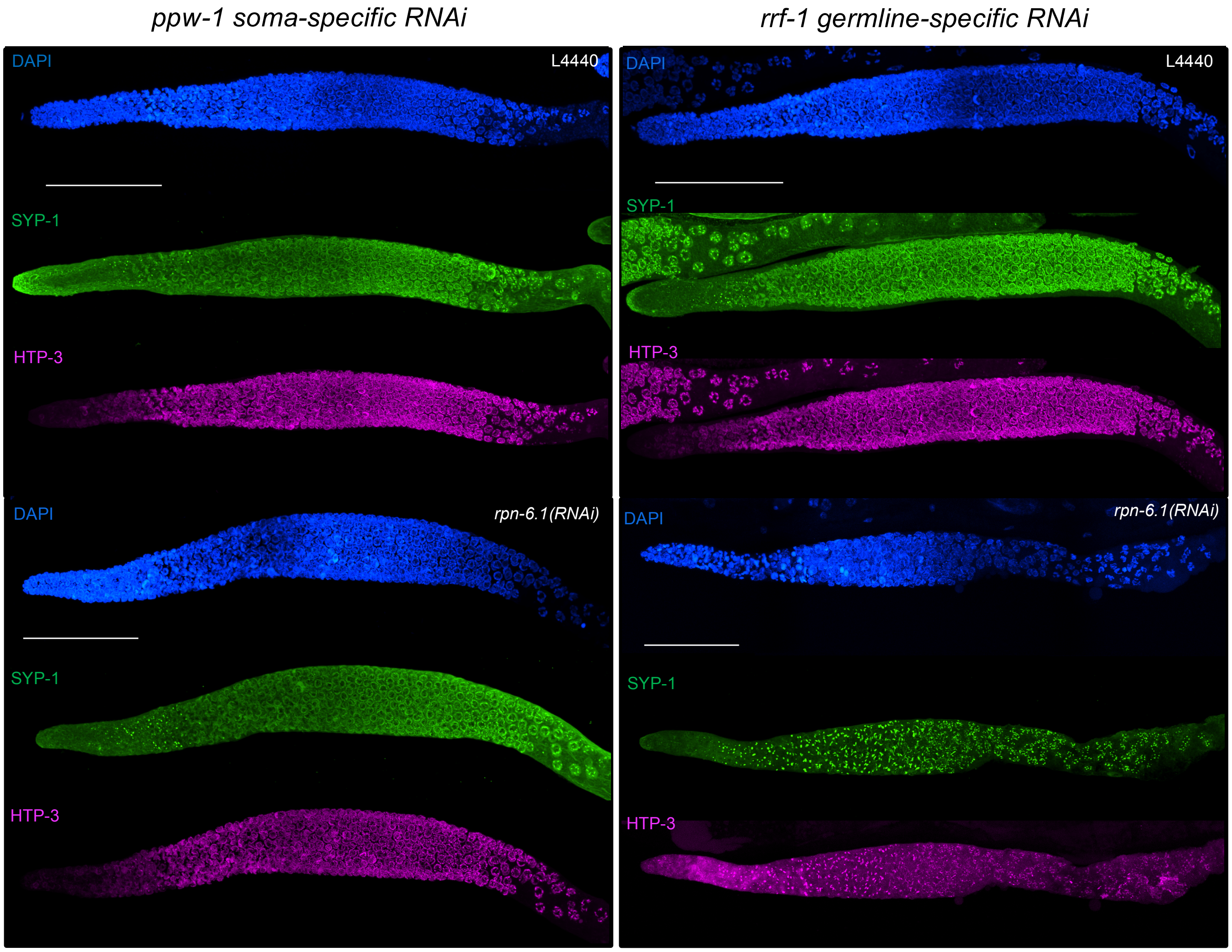

Supplemental Figure 7. Mitotic and SC defects due to proteasome knockdown are germline-autonomous. RNAi of proteasome subunits were performed on *ppw-1* and *rrf-1* strains and gonads were visualized after 48 hrs with DAPI (blue), anti-SYP-1 (green) to visualize SC protein, and anti-HTP-3 (magenta) to visualize the chromosome axis. Loss of proteasome function in the soma did not induce mitotic or SC defects, whereas loss in the germ line led to profound defects. Scale bar is 50μm.

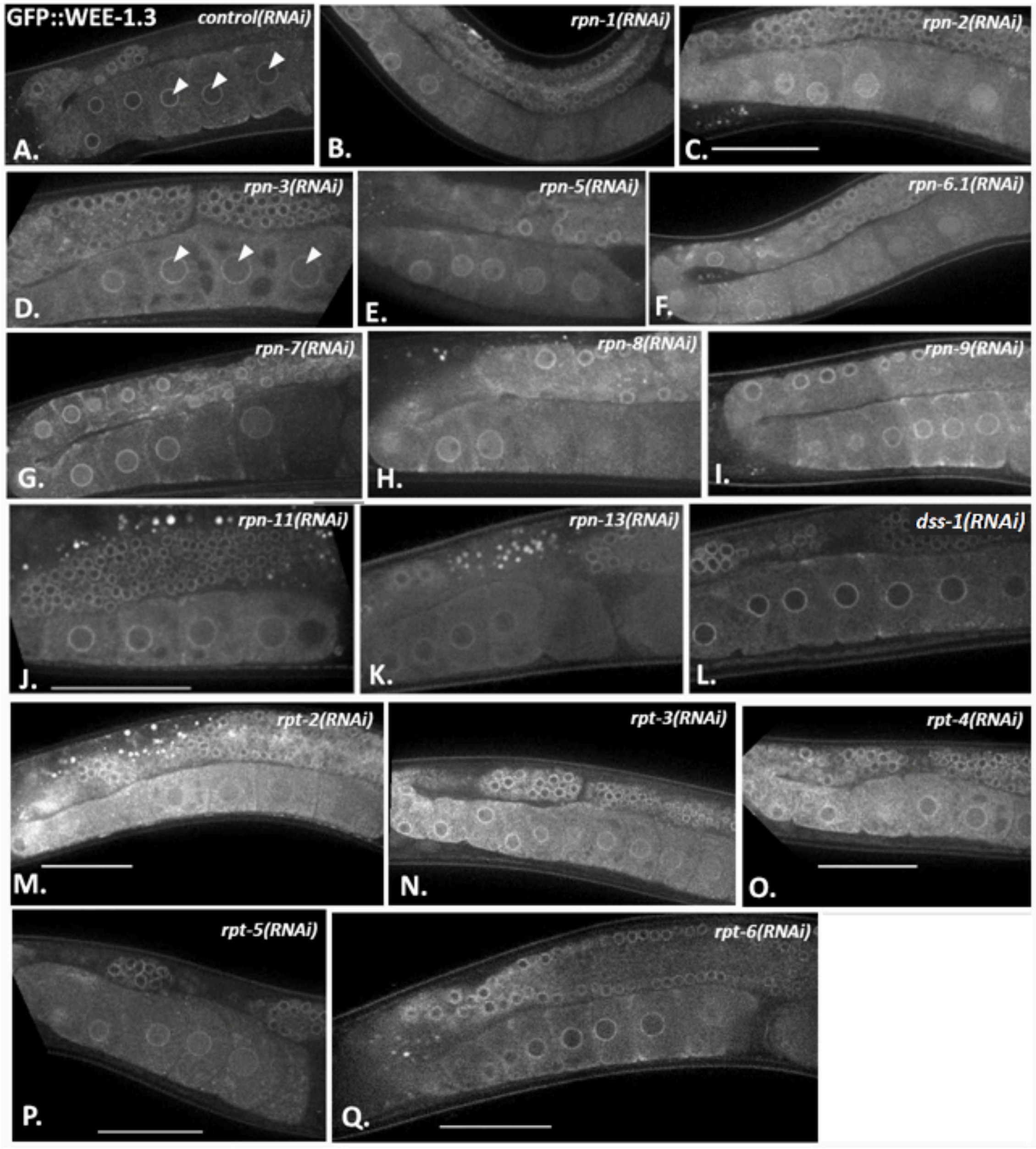

Supplemental Figure 8. WEE-1.3 function and localization are altered by depletion of specific proteasome subunits. Live imaging of gonads from strain WDC2 *wee-1.3(ana2[gfp::wee-1.3])* treated with the indicated RNAi condition. All images were taken at the same laser intensity and PMT gain. Scale bar is 100µm.

Supplemental Figure 9

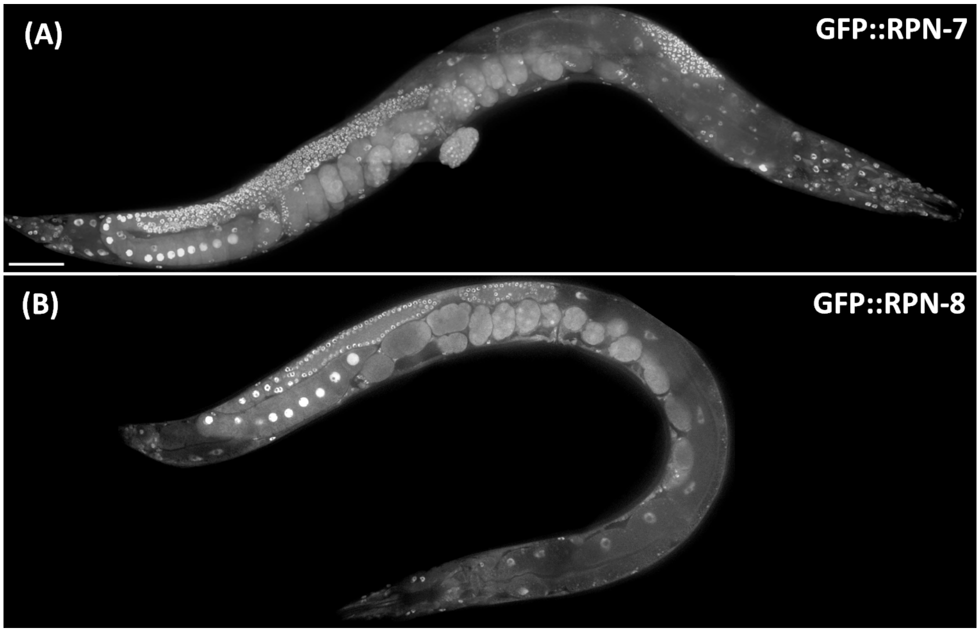

Supplemental Figure 9. GFP::RPN-7 and GFP::RPN-8 are ubiquitously expressed in the germ line and soma. Whole worm images of adult hermaphrodites expressing (A) GFP::RPN-7 (Max IP) and (B) GFP::RPN-8 (Single Z stack) exhibiting ubiquitous expression of RPN-7 and RPN-8 in the germ line tissues, soma and embryos with bright nuclear and relatively dim cytoplasmic expression. Scale bar represents 50μm.

Supplemental Figure 10

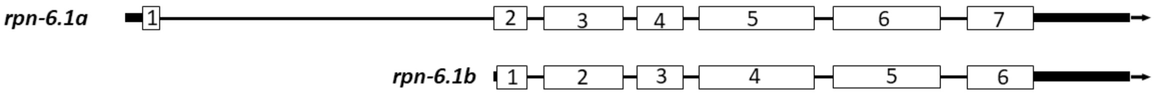

Supplemental Figure 10: Schematic of the two *C. elegans* protein isoforms for RPN-6.1. Boxes indicate exons while black lines indicate introns. Thick black lines indicate either 5' or 3' untranslated regions. RPN-6.1 isoform A (RPN-6.1A) contains an extra exon at the C-terminus of the protein, while RPN-6.1 isoform B (RPN-6.1B) is missing that first exon.

Supplemental Figure 11:

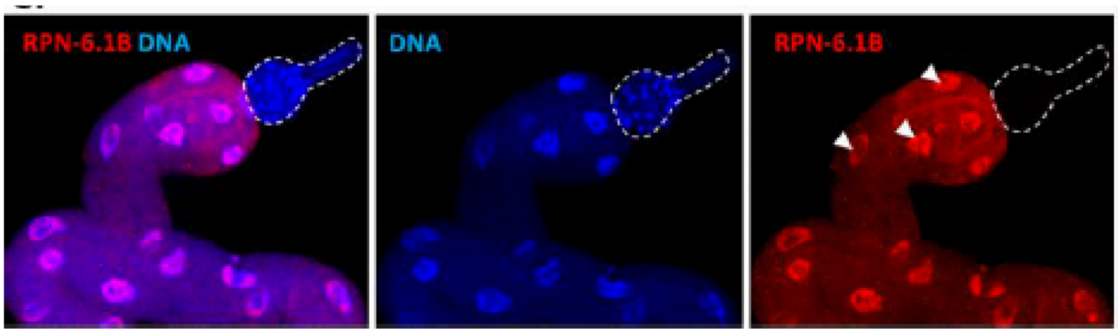

Supplemental Figure 11. The C-terminal tagged RPN-6.1::OLLAS exhibits expression in the intestine that is not observed with the N-terminal tagged GFP::RPN-6.1. Immunofluorescence image of *rpn-6.1(ana12[rpn-6.1::ollas])* strain dissected gonad co-stained with anti-OLLAS (red) and DAPI for DNA (blue). Bright nuclear and relatively dim cytoplasmic RPN-6.1b expression shown throughout the intestine. Interestingly, there is no RPN-6.1::OLLAS expression in the pharynx.

Supplemental Table 1: *C. elegans* proteasome subunits and their human and yeast orthologs. Includes a list of all the various 19S proteasome subunits in *C. elegans* with their number of predicted splice isoforms, size of the protein in kDA, chromosomal location, and the identified human and yeast ortholog.

* RPN-6.2 is a sperm-specific proteasome subunit (personal communication Dr. Lynn Boyd)

***C. elegans* proteasome subunit**	**Number of isoforms**	**Size (kDa)**	**Chromo -some**	**Human ortholog**	**Yeast ortholog**
RPN-1	1	107kDa	**IV**	PSMD2/S2	RPN1
RPN-2	2	a- 95.5kDa<br>b- 106kDa	**III**	PSMD1/S1	RPN2
RPN-3	1	57.5kDa	**III**	PSMD3/S3	RPN3
RPN-5	1	56.4kDa	**II**	PSMD12	RPN5
RPN-6.1	2	a- 49.1kDa<br>b- 47.1kDa	**III**	PSMD11/S9	RPN6
RPN-6.2*	2	a- 46.8kDa<br>b- 24.2kDa	**III**	PSMD11/S9	RPN6
RPN-7	1	47.6kDa	**IV**	PSMD6/S10	RPN7
RPN-8	1	40.7kDa	**I**	PSMD7/S12	RPN8
RPN-9	1	44.1kDa	**II**	PSMD13/S11	RPN9
RPN-10	1	37.3kDa	**I**	PSMD4/S5a	RPN10
RPN-11	1	34.6kDa	**II**	PSMD14/<br>Poh1/Pad1	RPN11
RPN-12	1	28.8kDa	**II**	PSMD8/S14	RPN12
RPN-13	2	a-39.8kDa<br>b-43.2kDa	**III**	ADRM1	RPN13
DSS-1 /<br>RPN-15	1	9.5kDa	**III**	PSMD9/<br>Dss1/ Rpn15	SEM1 /<br>DSS1
RPT-1	1	48.6kDa	**V**	PSMC2/S7	RPT1
RPT-2	1	49.7kDa	**V**	PSMC1/S1	RPT2
RPT-3	1	46.3kDa	**III**	PSMC4/S6	RPT3
RPT-4	2	a- 45.8kDa<br>b- 44.9kDa	**II**	PSMC6/S10	RPT4
RPT-5	1	48.1kDa	**I**	PSMC3/S6a	RPT5
RPT-6	1	46.2kDa	**III**	PSMC5/S8	RPT6

Supplemental Table 2. Strains list

Strain name	Description	Genotype	Source
NL3511	*ppw-1*	ppw-1 (pk1425) I	Tijsterman, et al., 2002
WDC1	*gfp::rpn-7*	rpn-7(ana1[gfp::rpn-7])	This study
WDC3	*gfp::rpn-6.1*	rpn-6.1(ana3[gfp::rpn-6.1])	This study
WDC4	*gfp::rpn-8*	rpn-8(ana4[gfp::rpn-8])	This study
WDC5	*gfp::rpn-9*	rpn-9(ana5[gfp::rpn-9])	This study
WDC6	*gfp::rpn-12*	rpn-12(ana6[gfp::rpn-12])	Fernando, et al., 2020
WDC12	*rpn-6.1::OLLAS*	rpn-6.1(ana12[rpn-6.1::OLLAS])	This study
NL2098	*rrf-1*	rrf'-1(pk1417) I	Sijen, et al., 2001
WDC2	*gfp::wee-1.3*	wee-1.3(ana2[gfp::wee-1.3])	Fernando, et al., 2020
IT1187	*UbG76V::GFP::H2B*	unc-119(ed3) III; kpIs100 [pie-1p::Ub(G76V)::GFP::H2B::drp-1 3' UTR; unc-119(+)]	Kumar, et al., 2018

Supplemental Table 3. crRNA sequences and properties

Gene	Strain	crRNA #	crRNA sequence 5'->3'	%GC	Distance*
rpn-6.1	*gfp::rpn-6.1*	crRNA18	gtgaactcgtttctctcatt	40	1bp
rpn-7	*gfp::rpn-7*	crRNA20	cattttaaggaggatgacag	40	1bp
rpn-8	*gfp::rpn-8*	crRNA21	acatctccgtctttgttagt	40	9bp
rpn-9	*gfp::rpn-9*	crRNA23	actgctcaagactacctcaa	45	17bp
wee-1.3	*gfp::wee-1.3*	crRNA29	gtgaaaatggacgacacaga	45	8bp
rpn-12	*gfp::rpn-12*	crRNA34	agccagaagatttttatggg	48	9bp
rpn-6.1	*rpn-6.1::OLLAS*	crRNA42	tacaattcatgcaatgggag	40	44bp

Supplemental Table 4. Primers to generate repair templates and diagnostics primers

**Strain**	**Silent mutations**	**Primers for repair oligo generation or ssODN and Diagnostics primers (5'→3')**
gfp::rpn-6.1	c→g	oAKA334 Fwd:<br>ttcaaaaaattattttaattgacacaacttttcgtgctaatgtccaagggagaggagctctt<br>oAKA335 Rev:<br>atattattagtgtcttctcgtgaactcgtttctctcttgtagagctcgtccattc<br>*Diagnostics*<br>oAKA386 Fwd: gttttgacatcctcgaagctg<br>oAKA387 Rev. cttcgttgtggttaaattgcac
gfp::rpn-7	g→a	oAKA338 Fwd:<br>atttcaagtgttcatatttcattttaaggaggatgtccaagggagaggagctctt<br>oAKA339 Rev:<br>tcatctacgggtttcttcgtgctctttttggcagcttctgtcttgtagagctcgtccattc<br>*Diagnostics*<br>oAKA388 Fwd: ggccgctttttaacgtttgc<br>oAKA389 Rev. gaacttgcggagtaatccct
gfp::rpn-8	c→t,<br>a→c,<br>g→t	oAKA340 Fwd:<br>ttgtgataaatttattttcgtttttttagaagaatgtccaagggagaggagctctt<br>oAKA341 Rev:<br>agaacatctacagctttgacagtcgccacatctccatcgttgttggttggagccttgtagagctcgtccattc<br>*Diagnostics*<br>oAKA392 Fwd: gcgtttctcactgttatgtcg<br>oAKA393 Rev: ccatgtcgaggaaccatgta
gfp::rpn-9	t→c,<br>a→c	oAKA344 Fwd:<br>ctcaatttttaatgtatcgagaatttctcaggatgtccaagggagaggagctctt<br>oAKA345 Rev:<br>ccattggcagccgccagctttccgttgaggtagtcgtgggcggtcttgtagagctcgtccattc<br>*Diagnostics*<br>oAKA394 Fwd: ccatgcgcctttaattgctg<br>oAKA395 Rev: gaaatgcaaatctcgacgagc
gfp::rpn-12	c→t	oAKA498 Fwd:<br>tatcaaattaaaacattattggatttaagaaaatgtccaagggagaggagctctt<br>oAKA499 Rev:<br>tcctttgcccacacagccagaagatttttatgggcagcagacttgtagagctcgtccattc<br>*Diagnostics*<br>oAKA415 Fwd: accttcacagaatcgtcgag<br>oAKA506 Rev: cattcaaatcgctggaggca
rpn-6.1	g→a	oAKA546 ssODN

::ollas		gtaccaaactgctctggatacaattcatgcaatgggagaagttgtcgatgcactct atagtaatgcttcgaaaattaactccggattcgccaacgagctcggaccacgtctc atgggaaagtgatgatttcttcgaatcttcatttttgttctgc<br>*Diagnostics*<br>oAKA521 Fwd: aggcgagggaatgcttattg<br>oAKA522 Rev: cgacgtattcctccgtgtat

20nt crRNA sequences are underlined.

Supplemental Table 5. Quantification of phospho-Histone H3 nuclei.

**Gene RNAi (n)**	**Number pH3+ nuclei/gonad**		
	**0 – 5**	**6 – 10**	**>10**
***rpn-2* (10)**	10%	20%	70%
***rpn-3* (10)**		40%	60%
***rpn-13* (8)**	62.5%	25%	12.5%
**WT (7)**	100%		

n = number of worms analyzed
